# Cutaneous melanoma: ESMO Clinical Practice Guideline for diagnosis, treatment and follow-up^⋆^

**DOI:** 10.1016/j.annonc.2024.11.006

**Published:** 2024-11-14

**Authors:** T. Amaral, M. Ottaviano, A. Arance, C. Blank, V. Chiarion-Sileni, M. Donia, R. Dummer, C. Garbe, J. E. Gershenwald, H. Gogas, M. Guckenberger, J. Haanen, O. Hamid, A. Hauschild, C. Höller, C. Lebbé, R. J. Lee, G. V. Long, P. Lorigan, E. Muñoz Couselo, P. Nathan, C. Robert, E. Romano, D. Schadendorf, V. Sondak, K. P. M. Suijkerbuijk, A. C. J. van Akkooi, O. Michielin, P. A. Ascierto, on behalf of the ESMO Guidelines Committee

**Affiliations:** 1 Skin Cancer Clinical Trials Center— University of Tuebingen, Tuebingen, Germany; 2 Department of Melanoma, Cancer Immunotherapy and Development Therapeutics, Istituto Nazionale Tumori IRCCS Fondazione “G. Pascale”, Napoli, Italy; 3 Department of Medical Oncology and IDIBAPS, Hospital Clinic y Provincial de Barcelona, Barcelona, Spain; 4 Department of Medical Oncology and Division of Immunology, The Netherlands Cancer Institute Antoni van Leeuwenhoek Ziekenhuis (NKI), Amsterdam; 5 Leiden University Medical Center (LUMC), Leiden, The Netherlands; 6 University Clinic Regensburg, Regensburg, Germany; 7 Department of Oncology, Melanoma Unit, Istituto Oncologico Veneto, IOV- IRCCS, Padova, Italy; 8 National Center for Cancer Immune Therapy (CCIT-DK), Department of Oncology, Copenhagen University Hospital Herlev and Gentofte, Herlev, Denmark; 9 Department of Dermatology, Skin Cancer Center, USZ—University Hospital Zürich, University of Zürich, Zürich, Switzerland; 10 Department of Dermatology, Center for DermatoOncology, University Hospital Tuebingen, Tuebingen, Germany; 11 Department of Surgical Oncology, The University of Texas MD Anderson Cancer Center and The University of Texas MD Anderson Cancer Center UTHealth Houston Graduate School of Biomedical Sciences, Houston, USA; 12 First Department of Medicine, School of Medicine, National and Kapodistrian University of Athens—School of Medicine, Athens, Greece; 13 Department of Radiation Oncology, University Hospital Zürich, University of Zürich, Zürich, Switzerland; 14 Division of Medical Oncology, Netherlands Cancer Institute, Amsterdam; 15 Department of Medical Oncology, Leiden University Medical Center (LUMC), Leiden, The Netherlands; 16 Oncology Service, Melanoma Clinic, Centre Hospitalier Universitaire Vaudois, Lausanne, Switzerland; 17 Medical Oncology, Cutaneous Malignancies, The Angeles Clinic and Research Institute, A Cedars Sinai Affiliate, Los Angeles, USA; 18 Department of Dermatology, UKSH—Universitätsklinikum Schleswig-Holstein—Campus Kiel, Kiel, Germany; 19 Department of Dermatology, Medical University of Vienna, Vienna, Austria; 20 Université Paris Cite, AP-HP Dermato-oncology and CIC, Cancer Institute APHP, Nord Paris Cité, INSERM U976, Saint Louis Hospital, Paris, France; 21 Department of Medical Oncology, The Christie NHS Foundation Trust, Manchester; 22 Faculty of Biology Medicine and Health, The University of Manchester, Manchester, UK; 23 Melanoma Institute Australia, The University of Sydney, Sydney; 24 Department Medical Oncology, Royal North Shore Hospital, Sydney; 25 Mater Hospital, Sydney, Australia; 26 Department of Medical Oncology, Vall d’Hebron University Hospital and Vall d’Hebron Institute of Oncology (VHIO), Barcelona, Spain; 27 Mount Vernon Cancer Centre, Northwood, UK; 28 Department of Oncology, Institut Gustave Roussy and Paris-Saclay University, Villejuif; 29 Department of Oncology, Center for Cancer Immunotherapy, Institut Curie, Paris, France; 30 Department of Dermatology, WTZ—Westdeutsches Tumorzentrum Essen, National Center for Tumor Diseases (NCT-West), Campus Essen, Essen; 31 University Alliance Ruhr, Research Center One Health, University Duisburg-Essen, Essen, Germany; 32 Department of Cutaneous Oncology, Moffitt Cancer Center, Tampa, USA; 33 Department of Medical Oncology, University Medical Center Utrecht, Utrecht University, Utrecht, The Netherlands; 34 Department of Melanoma and Surgical Oncology, Royal Prince Alfred Hospital, Sydney, Australia; 35 Department of Oncology, Geneva University Hospital, Geneva, Switzerland; 36 Melanoma, Cancer Immunotherapy and Development Therapeutics Unit, Instituto Nazionale Tumori IRCCS Fondazione Pascale, Napoli, Italy

**Keywords:** cutaneous melanoma, diagnosis, ESMO Clinical Practice Guideline, prognosis, risk assessment, treatment

## Introduction

This European Society for Medical Oncology (ESMO) Clinical Practice Guideline (CPG) focuses on invasive cutaneous melanoma. The management of uveal melanoma and non-melanoma skin cancers are described in separate ESMO CPGs. Mucosal melanoma is described in the Supplementary Material Section 1 and Table S1, available at https://doi.org/10.1016/j.annonc.2024.11.006.

## Incidence and Epidemiology

Details on incidence and epidemiology of cutaneous melanoma are provided in the Supplementary Material Section 2, available at https://doi.org/10.1016/j.annonc.2024.11.006.

## Diagnosis and Pathology/Molecular Biology

### Diagnosis

Details on the diagnostic work-up of cutaneous melanoma are provided in the Supplementary Material Section 3 and Tables S2 and S3, and a proposed algorithm is shown in Supplementary Figure S1, available at https://doi.org/10.1016/j.annonc.2024.11.006.

### Molecular characterisation

Testing for actionable mutations is recommended for patients with resectable or unresectable stage III or IV melanoma. Mutation testing should be considered for high-risk, clinical stage IIB-IIC melanoma, but is not routinely recommended for stage I or IIA disease. Mutation testing of *BRAF* V600 is mandatory, whereas testing for other *BRAF* mutations is optional. A full list of *BRAF* mutations by class is provided in Supplementary Table S4, available at https://doi.org/10.1016/j.annonc.2024.11.006. Testing can be offered for *NRAS* and *c-KIT* mutations; testing for *NTRK* alterations is recommended in the absence of *BRAF* or *RAS* mutations [see ESMO Scale for Clinical Actionability of molecular Targets (ESCAT) for further details—Supplementary Table S5, available at https://doi.org/10.1016/j.annonc.2024.11.006]. Mutation analysis using next-generation sequencing can be offered for unresectable melanoma. Mutation analysis must be carried out in accredited (certified) institutes that have careful quality controls and appropriate bioinformatic knowledge.

The main melanoma subtypes are associated with different mutational landscapes,^1^ as shown in Supplementary Table S6, available at https://doi.org/10.1016/j.annonc.2024.11.006. In addition to the mutational status, reporting programmed death-ligand 1 (PD-L1) expression by immunohistochemistry is recommended for all unresectable stage III and IV melanoma, since the European Medicines Agency (EMA) has approved the administration of nivolumab–relatlimab only in patients with tumour cell PD-L1 expression <1%.^2^ Tumour mutational burden computed on full exome sequencing or on a large full-length panel and expressed as the number of mutations per megabase can be assessed and recorded, but its clinical use is currently not warranted.^1^

Signatures combining different gene panels and clinical/pathological characteristics (e.g. AMBLor,^3^ DecisionDx-Melanoma,^4^ Immunoprint,^5^ MelaGenix,^4^ SkylineDx^6^) have shown promising prognostic value in primary cutaneous melanoma. However, current evidence does not support the use of these tests in routine clinical practice. Prospective studies are underway to assess their predictive and prognostic value, which will provide valuable insights into their potential future clinical utility.^7^

### Recommendations

- Dermoscopy by an experienced physician is recommended and increases the diagnostic accuracy [II, A].
- Diagnosis should be based on a full thickness complete excision with a minimal margin of clinically uninvolved skin [II, A]. For larger lesions where complete excision is not possible without reconstructive surgery, a biopsy can be taken [II, C].
- The histology report should include at least information on the type of melanoma, maximum vertical tumour thickness in millimetres (Breslow, measured to the nearest 0.1 mm), presence of ulceration, microsatellites, lymphovascular invasion, neurotropism/perineural invasion, tumour-infiltrating lymphocytes (TILs), presence of regression and presence or absence of tumour at the deep and peripheral edges of the biopsy [II, A]. Mitotic rate should also be reported [III, B].
- A report on the wide excision should also be made available for complete pathological characterisation [II, A].
- Testing for actionable mutations is recommended in patients with resectable or unresectable stage III or IV melanoma [I, A] and should be considered in clinical stage IIB-IIC [V, C] but not for stage I or IIA disease [V, D].
  ○ *BRAF* V600 testing is mandatory [I, A; ESCAT score: I-A].

## Staging and Risk Assessment

Details on the staging and risk assessment of cutaneous melanoma are provided in the Supplementary Material Section 4 and Table S2, available at https://doi.org/10.1016/j.annonc.2024.11.006.

### Recommendations

- Staging should be according to the eighth edition of the American Joint Committee on Cancer (AJCC) TNM (tumour–node–metastasis) staging system (AJCC8) [II, A].
- Sentinel lymph node biopsy (SLNB) is not routinely recommended for patients with a melanoma of AJCC8 stage pT1a (e.g. with a tumour thickness <0.8 mm and no ulceration) [II, E].
- SLNB is not usually recommended but can be discussed in pT1a for special cases [e.g. 3 mitoses/mm^2^, a positive deep margin or when Breslow thickness cannot be reliably determined (pTx)] [III, D].
- SLNB should be discussed with patients with a melanoma of AJCC8 stage pT1b (i.e. with a tumour thickness of 0.8-1.0 mm or with a tumour thickness of <0.8 mm with ulceration) [III, B].
- SLNB is recommended for all patients with clinically node-negative T2a or higher tumours according to AJCC8 criteria (>1.0 mm Breslow thickness) [I, A]. Whenever possible, wide excision of the primary tumour should be carried out at the same time.
- For patients with T3b, T4a and T4b melanoma who qualify for adjuvant therapy, omitting SLNB can be discussed with the patient, but the potential benefits of SLNB in terms of staging (especially in patients with a *BRAF*-mutated, thick primary stage IIB or IIC who could be upstaged to stage III melanoma) and locoregional control should also be discussed [V, C].
- Whole-body physical examination with special attention to the primary tumour residual intact component and other suspicious pigmented cutaneous lesions, tumour satellites, in-transit metastases (ITMs) and regional lymph nodes (LNs) is recommended [IV, A]. In tumour stages IIB or higher, ultrasound (US), computed tomography (CT) and/or positron emission tomography (PET) scans and brain magnetic resonance imaging (MRI) are recommended to ensure proper tumour assessment [III, B].

## Management of Local/Locoregional Disease

### Treatment of localised melanoma

Full depth, wide local excision (WLE) of primary tumours (with safety margins of 0.5 cm for *in situ* melanomas, 1 cm for tumours with a thickness of ≤2 mm and 2 cm for tumours thicker than 2 mm) is recommended (see Supplementary Table S7, available at https://doi.org/10.1016/j.annonc.2024.11.006).^8^ Modifications, with reduced safety margins, or micrographic surgery, are acceptable for preservation of function in acral and facial melanomas, including lentigo maligna melanoma.

Definitive radiotherapy (RT) to the primary tumour can only be considered in carefully selected patients for local control when excision is not possible either due to severe patient comorbidities (e.g. very old age, end-stage cardiovascular disease, etc.), when the morbidity associated with the excision is considered unacceptable or when surgery is refused by the patient. Palliative RT and palliative surgery can be offered for local control of symptomatic lesions.

### Treatment of locoregional melanoma

**Figure 1:**
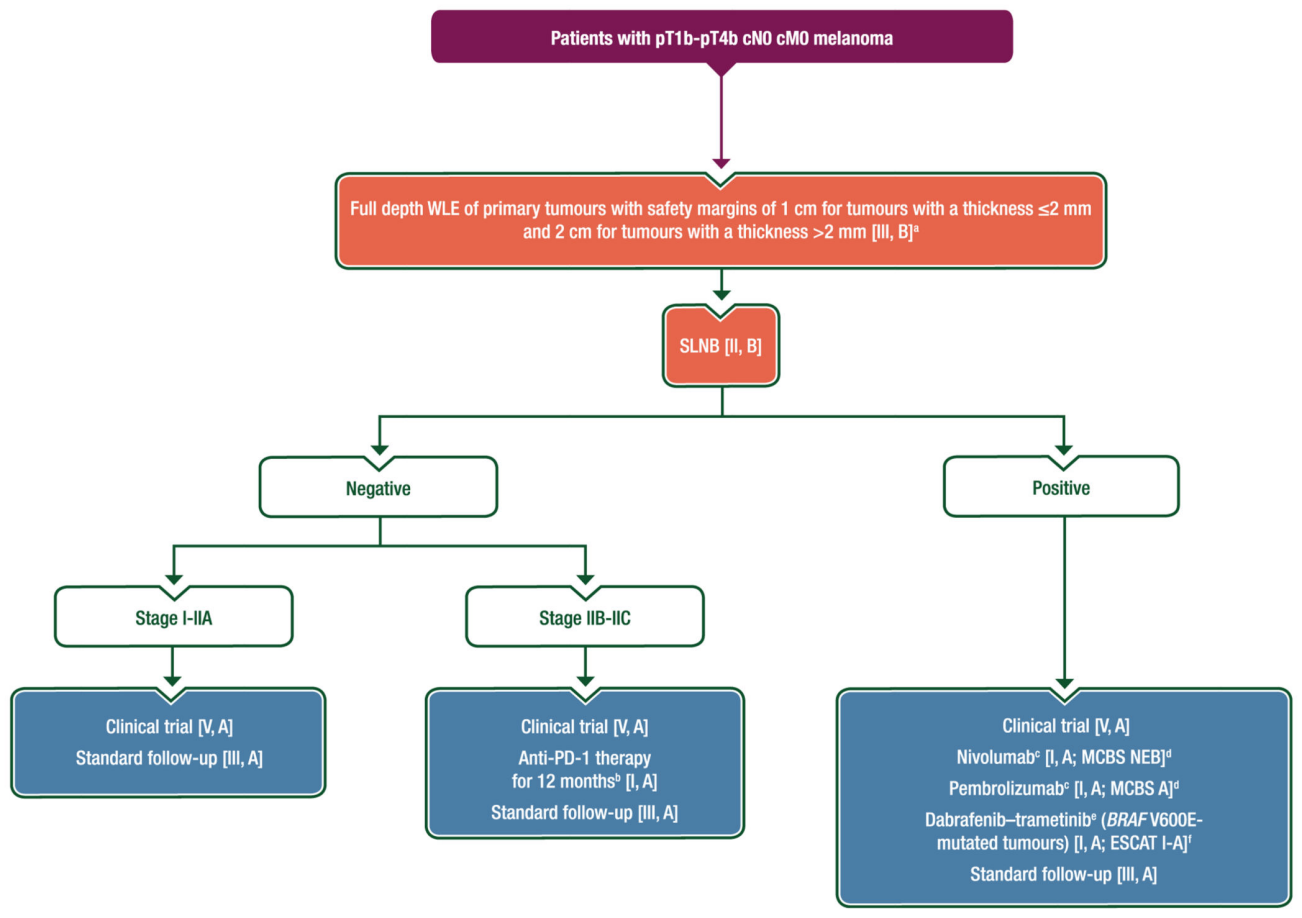
Proposed algorithm for the management of patients with pT1b-pT4b cN0 cM0 melanoma. Purple: algorithm title; blue: systemic anticancer therapy; orange: surgery; white: non-treatment aspects. c, clinical; DMFS, distant metastasis-free survival; EMA, European Medicines Agency; ESCAT, ESMO Scale for Clinical Actionability of molecular Targets; ESMO, European Society for Medical Oncology; FDA, Food and Drug Administration; LM, lentigo maligna; M, metastasis; MCBS, ESMO-Magnitude of Clinical Benefit Scale; N, node; NEB, no evaluable benefit; OS, overall survival; p, pathological; PD-1, programmed cell death protein 1; R1, microscopic tumour at the margin; RFS, recurrence-free survival; RT, radiotherapy; SLNB, sentinel lymph node biopsy; T, tumour; WG, working group; WLE, wide local excision. ^a^RT can be considered for local tumour control in cases of inadequate resection margins of LM [III, B] and could be discussed for patients with an R1 resection [III, C]. Adjuvant RT to the primary excision site should be considered for patients with desmoplastic or neurotropic melanoma for whom adequate (≥8 mm) pathological resection margins cannot be achieved [IV, C]. ^b^Treatment discussions with the patient should include consideration of the RFS benefit but lack of mature OS data [I, A]. ^c^Treatment discussions with the patient should consider the DMFS and RFS benefits but lack of mature OS data compared with placebo [I, A]. ^d^ESMO-MCBS v1.1^78^ was used to calculate scores for therapies/indications approved by the EMA or FDA. The scores have been calculated and validated by the ESMO-MCBS WG and reviewed by the authors (https://www.esmo.org/guidelines/esmo-mcbs/esmo-mcbs-evaluation-forms). ^e^Treatment discussions with the patient should consider the DMFS and RFS benefits and potential OS benefit for patients with *BRAF* V600E-mutated melanoma [I, A]. ^f^ESCAT scores apply to genomic alterations only. These scores have been defined by the guideline authors and assisted as needed by the ESMO Translational Research and Precision Medicine Working Group.^77^

**Figure 2:**
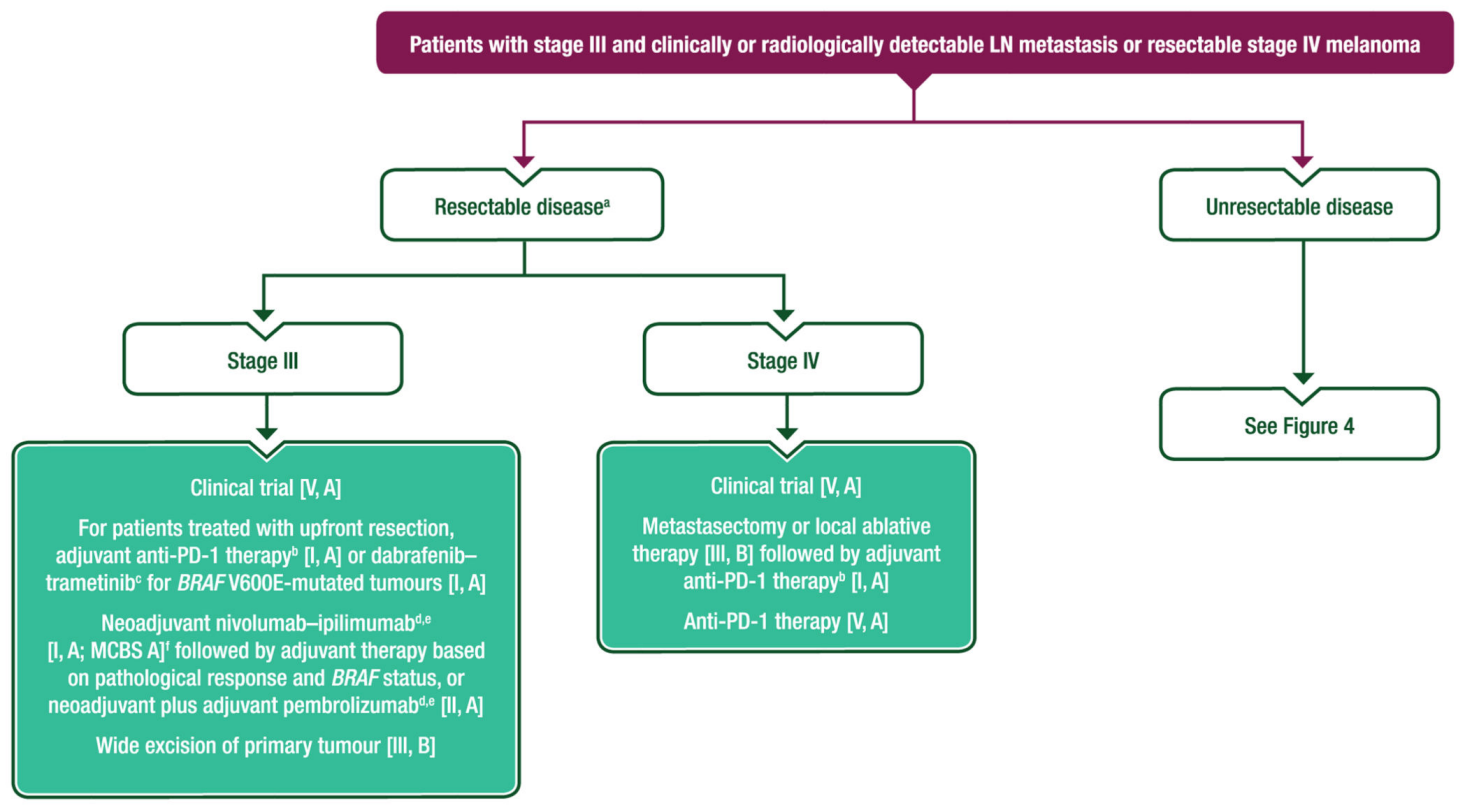
Proposed algorithm for the management of patients with stage III melanoma and clinically positive LNs or resectable stage IV melanoma. Purple: algorithm title; turquoise: combination of treatments and treatment modalities; white: non-treatment aspects. DMFS, distant metastasis-free survival; EMA, European Medicines Agency; ESMO, European Society for Medical Oncology; FDA, Food and Drug Administration; LN, lymph node; MCBS, ESMO-Magnitude of Clinical Benefit Scale; OS, overall survival; PD-1, programmed cell death protein 1; RFS, recurrence-free survival; RT, radiotherapy; WG, working group. ^a^RT could be discussed for patients after resection of bulky LN metastases, especially if further surgical clearance is not feasible [III, C]. ^b^Treatment discussions with the patient regarding adjuvant anti-PD-1 therapy should consider the DMFS and RFS benefits but lack of mature OS data compared with placebo [I, A]. ^c^Treatment discussions with the patient regarding adjuvant targeted therapy should consider the DMFS and RFS benefits and potential OS benefit for patients with *BRAF* V600E-mutated melanoma [I, A]. ^d^Not EMA or FDA approved as neoadjuvant therapy. ^e^Treatment discussions with the patient regarding neoadjuvant therapy should consider the EFS, DMFS and RFS benefits but lack of mature OS data [I, A]. ^f^ESMO-MCBS v1.1^78^ was used to calculate scores for therapies/indications approved by the EMA or FDA. The scores have been calculated and validated by the ESMO-MCBS WG and reviewed by the authors (https://www.esmo.org/guidelines/esmo-mcbs/esmo-mcbs-evaluation-forms).

Proposed treatment algorithms for the primary treatment of locoregional melanoma are shown in Figures 1 and 2.

For patients with a positive SLNB, complete LN dissection (CLND) or irradiation of regional LNs should not be carried out.^9–12^ Before undertaking additional treatments, a detailed staging investigation that includes high-resolution imaging techniques, such as CT, PET/CT and brain MRI, is necessary to exclude distant metastases.^13^

### Treatment of ITMs

**Figure 3:**
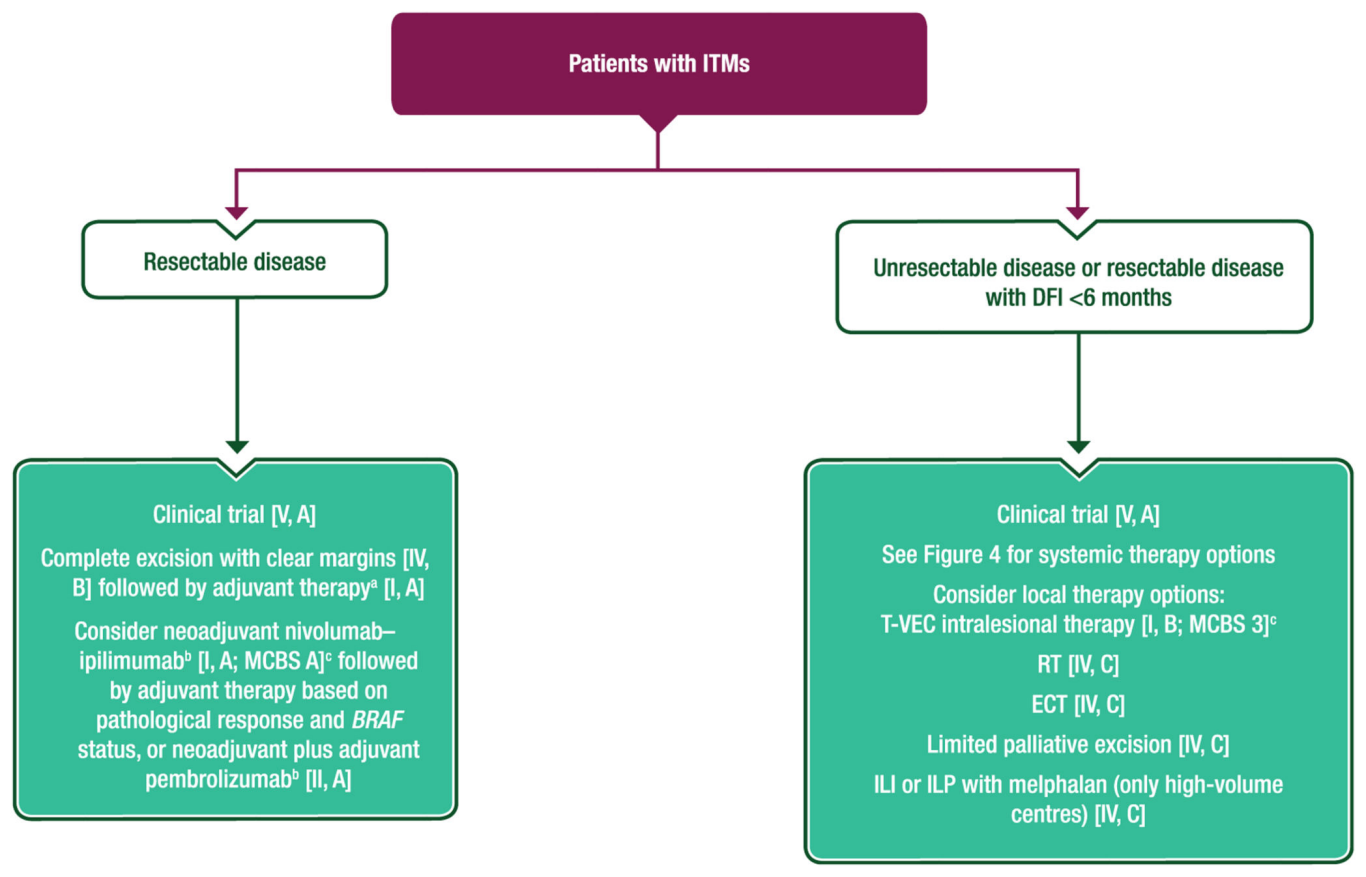
Proposed algorithm for the management of patients with ITMs. Purple: algorithm title; turquoise: combination of treatments and treatment modalities; white: non-treatment aspects. DFI, disease-free interval; DMFS, distant metastasis-free survival; ECT, electrochemotherapy; EMA, European Medicines Agency; ESMO, European Society for Medical Oncology; FDA, Food and Drug Administration; ILI, isolated limb infusion; ILP, isolate limb perfusion; ITM, in-transit metastasis; MCBS, ESMO-Magnitude of Clinical Benefit Scale; OS, overall survival; PD-1, programmed cell death protein 1; RFS, recurrence-free survival; RT, radiotherapy; T-VEC, talimogene laherparepvec; WG, working group. ^a^For anti-PD-1-based therapy, treatment discussions with the patient should consider the DMFS and RFS benefits but lack of mature OS data compared with placebo [I, A]. For dabrafenibetrametinib, these discussions should also consider the DMFS and RFS benefits and potential OS benefit for patients with *BRAF* V600E-mutated melanoma [I, A]. ^b^Not EMA or FDA approved as neoadjuvant therapy. ^c^ESMO-MCBS v1.1^78^ was used to calculate scores for therapies/indications approved by the EMA or FDA. The scores have been calculated and validated by the ESMO-MCBS WG and reviewed by the authors (https://www.esmo.org/guidelines/esmo-mcbs/esmo-mcbs-evaluation-forms).

A proposed algorithm for the management of ITMs is provided in Figure 3.

Patients with resectable disease but a short disease-free interval (<6 months), unresectable satellite, ITMs or inoperable primary tumours of the limbs without additional metastases should be treated with systemic therapy, preferentially anti-programmed cell death protein 1 (PD-1)-based therapy, as these patients are at risk for developing distant metastasis. Treatment of these patients should be discussed at a multidisciplinary tumour board.

### Adjuvant RT

Details regarding adjuvant RT are provided in Supplementary Material Section 5, available at https://doi.org/10.1016/j.annonc.2024.11.006.

### Adjuvant immunotherapy for resectable stage II-IV disease

A summary of results from key trials in the adjuvant setting is shown in Supplementary Table S8, available at https://doi.org/10.1016/j.annonc.2024.11.006.

#### Stage IIB-IIC disease

Considering the 10-year melanoma-specific survival (MSS) rate in stage IIB-IIC disease, and the high risk of recurrence in these patients,^14,15^ two trials investigating adjuvant therapy, with a primary endpoint of recurrence-free survival (RFS), were conducted.

In the KEYNOTE-716 trial, 976 patients (age ≥12 years) with completely resected AJCC8 pathological stage IIB-IIC melanoma received intravenous pembrolizumab 200 mg (2 mg/kg in paediatric patients) or placebo every 3 weeks (q3w) for 17 cycles or until disease recurrence or unacceptable toxicity. The estimated 36-month RFS rate was 76.2% for pembrolizumab and 63.4% for placebo [hazard ratio (HR) 0.62, 95% confidence interval (CI) 0.49-0.79], and the estimated 36-month distant metastasis-free survival (DMFS) rate was 84.4% for pembrolizumab versus 74.7% for placebo (HR 0.59, 95% CI 0.44-0.79).^16^ In June 2022, the EMA approved the use of adjuvant pembrolizumab for patients ≥12 years old with stage IIB-IIC melanoma following complete resection.

In the CheckMate 76K trial, patients with AJCC8 pathological stage IIB-IIC melanoma (i.e. similar to KEYNOTE-716 eligibility criteria) were randomised to receive either adjuvant nivolumab or placebo for up to 1 year or until disease recurrence or unacceptable toxicity. At a median follow-up of ~23 months, adjuvant nivolumab improved RFS versus placebo (HR 0.53, 95% CI 0.40-0.71). Higher RFS rates were observed for nivolumab regardless of disease stage or T category.^17^ The licensed indication for nivolumab as adjuvant therapy has been expanded to include patients with stage IIB-IIC melanoma.

Currently, no overall survival (OS) data are available from the KEYNOTE-716 and CheckMate 76K trials.

Based on these findings, clinicians can offer adjuvant anti-PD-1 treatment for patients with AJCC8 stage IIB-IIC disease after a detailed discussion with the patient to weigh the pros and cons of treatment benefit versus toxicity and a careful evaluation of clinical factors, including patient age, comorbidities, performance status (PS), reproductive potential, personal/family history of autoimmune disease and compliance in case of immune-related toxicity.

#### Resectable stage III disease

It is worth noting that entry criteria for most adjuvant trials in this setting were based on the AJCC seventh edition (AJCC7),^18^ and that stage IIIA disease defined by AJCC7 includes a higher-risk group of patients than stage IIIA defined by AJCC8, which also incorporates Breslow thickness into stage III disease (5-year MSS rates for AJCC7 versus AJCC8 stage IIIA disease are 78% versus 93%, respectively).^15^ Moreover, patients with AJCC7 stage IIIA disease were either excluded (CheckMate 238) or had to have >1 mm tumour metastasis from the SLNB to be included. Therefore, for patients with AJCC8 stage IIIA and <1 mm SLNB tumour burden, in the absence of prospective validation of the benefit of adjuvant therapy in this patient population, adjuvant therapy should not be considered as the standard treatment.^19^

Efficacy and safety data from prospective randomised controlled trials evaluating adjuvant treatment with immune checkpoint inhibitors (ICIs; ipilimumab, nivolumab, pembrolizumab) in patients with high-risk resected stage III melanoma are summarised below. It is important to note that the eligibility criteria for all trials except CheckMate 915 included complete resection of all disease, including primary tumour excision with adequate margins and CLND in patients with nodal metastases detected by SLNB and those with clinical evidence of regional disease. Although it is unclear if the recommended adjuvant treatment options have similar efficacy in the absence of CLND following a positive SLNB, the consistent 24-month RFS rates reported for nivolumab in CheckMate 238 and CheckMate 915^20^ suggest that CLND should not be a factor in the decision to use adjuvant therapy in patients with nodal metastases detected by SLNB.

#### Anti-cytotoxic T lymphocyte-associated antigen 4

In the EORTC 18071 trial,^21^ long-term adjuvant therapy with the anti-cytotoxic T lymphocyte-associated antigen 4 (CTLA-4) agent ipilimumab resulted in improved 5-year RFS (HR 0.76, 95% CI 0.64-0.89, *P* < 0.001) and OS rates (HR 0.72, 95.1% CI 0.58-0.88, *P* = 0.001) compared with placebo. The benefit was also observed for patients with N1b and higher disease stages. However, the treatment schedule (10 mg/kg q3w for four doses and then every 3 months for up to 3 years) was associated with several severe and long-lasting adverse reactions and is no longer used. Given the toxicity profile of anti-CTLA-4 and the duration of therapy investigated, adjuvant therapy with either anti-PD-1 agents or dabrafenib–trametinib for patients with *BRAF*-mutated melanoma is preferred.

#### Anti-PD-1

In the CheckMate 238 trial, adjuvant therapy with the anti-PD-1 agent nivolumab has shown a significant RFS benefit^22^ but no significant OS benefit compared with ipilimumab 10 mg/kg for patients with stage IIIB, IIIC or IV (AJCC7) resected melanoma with no evidence of disease (NED) (see details in ‘Resectable stage III and IV NED’ section below).^23^

Adjuvant treatment with the anti-PD-1 agent pembrolizumab was evaluated in patients with AJCC7 stage IIIA (SLN >1 mm), IIIB or IIIC (without ITM) melanoma in the placebo-controlled EORTC 1325/KEYNOTE-054 trial. At a median follow-up of 4.9 years, the 5-year RFS rate was longer in the pembrolizumab group than in the placebo group in the intention-to-treat population (HR 0.61, 95% CI 0.51-0.72) and in those with PD-L1-positive tumours (HR 0.62, 95% CI 0.48-0.79). Moreover, the 5-year DMFS rate was higher in the pembrolizumab group than in the placebo group (HR 0.62, 95% CI 0.52-0.75).^24^ OS data are not yet available.

These results were validated in the phase III S1404 trial, which compared adjuvant pembrolizumab with either of the two standard-of-care (SoC) options at the time (i.e. high-dose interferon-α or ipilimumab 10 mg/kg). RFS was significantly longer in the pembrolizumab group than in the SoC group (HR 0.76, 99.62% CI 0.59-0.99, log-rank *P=* 0.002).^25^

Based on these data, the EMA approved nivolumab and pembrolizumab for use in the adjuvant setting in August and December 2018, respectively.

#### Resectable stage III and IV NED

In the CheckMate 238 trial,^23^ which compared nivolumab with ipilimumab 10 mg/kg in patients with AJCC7 stage IIIB, IIIC or IV resected melanoma, the 5-year RFS rates were 50% in the nivolumab group and 39% in the ipilimumab group (HR 0.72, 95% CI 0.60-0.86). The 5-year RFS rates according to disease stage (IIIB, IIIC, IV M1a-b, IV M1c) were 58%, 43%, 47% and 55% for nivolumab and 48%, 35%, 29% and 49% for ipilimumab. The 5-year DMFS and OS rates were 58% versus 51% (HR 0.79, 95% CI 0.63-0.99) and 76% versus 72% (HR 0.86, 95% CI 0.66-1.12), respectively.^22^ Moreover, nivolumab treatment was associated with fewer grade 3/4 adverse events (AEs) compared with ipilimumab (14.4% versus 45.9%, respectively).^23^

In the IMMUNED phase II trial, 167 patients with stage IV melanoma and NED after surgery or RT were randomised to receive nivolumab 1 mg/kg plus ipilimumab 3 mg/kg q3w for four doses followed by nivolumab 3 mg/kg every 2 weeks (q2w), nivolumab monotherapy (3 mg/kg q2w) or matching placebo for up to 1 year. The HR for RFS for nivolumab–ipilimumab versus placebo was 0.25 (97.5% CI 0.13-0.48, *P* < 0.0001) and for nivolumab versus placebo was 0.60 (97.5% CI 0.36-1.00, *P* = 0.024). The HR (95% CI) for RFS according to disease stage (M1a-b and M1c) for nivolumab–ipilimumab versus placebo was 0.29 (0.15-0.55) and 0.18 (0.06-0.57), and for nivolumab versus placebo was 0.50 (0.29-0.86) and 0.87 (0.40-1.90). The HR for OS was significantly in favour of nivolumab–ipilimumab versus placebo (HR 0.41, 95% CI 0.17-0.99, *P* = 0·040) but not for nivolumab versus placebo (HR 0.75, 95% CI 0.36-1.56, *P* = 0.44). Rates of grade 3/4 treatment-related AEs (TRAEs) were numerically higher with combination therapy, but types of toxicity were similar to what is already known for these agents.^26^

CheckMate 915 was a phase III, double-blind trial in resected stage IIIB-IIID or IV melanoma. Patients were randomised to receive nivolumab 240 mg q2w plus ipilimumab 1 mg/kg once every 6 weeks or nivolumab 480 mg every 4 weeks (q4w) for ≤1 year. There was no significant difference between treatment groups reported for median RFS in the all-randomly assigned patient population (HR 0.92, 95% CI 0.77-1.09, *P=* 0.269) or in patients with PD-L1 tumour expression <1% (HR 0.91, 95% CI 0.73-1.14), or in 2-year RFS rates according to disease stage (IIIB: HR 0.91, 95% CI 0.68-1.21; IIIC: HR 0.92, 95% CI 0.75-1.13; IIID: HR 1.61, 95% CI 0.70-3.67; IV: HR 0.88, 95% CI 0.58-1.32).^20^

Currently, adjuvant treatment with nivolumab–ipilimumab is included as a treatment option in the National Comprehensive Cancer Network guideline for cutaneous melanoma in patients with stage IV melanoma and NED,^27^ but this adjuvant treatment combination is not approved by the EMA or Food and Drug Administration (FDA).

Other systemic therapies are currently being evaluated as adjuvant therapy for patients with resected stage IIB-IV melanoma. In an ongoing phase II clinical trial, the addition of messenger RNA-4157 to adjuvant pembrolizumab has shown a 49% risk reduction in recurrence and/or death (HR 0.510, 95% CI 0.288-0.906, two-sided nominal *P* = 0.019) versus pembrolizumab alone.^28^ A phase III trial is also ongoing.

### Adjuvant targeted therapy for resectable stage II-III disease

The BRIM8 study evaluated single-agent vemurafenib versus placebo in patients with stage IIC and stage III (AJCC7 criteria) melanoma after complete surgical resection. The study did not meet its primary endpoint of DFS.^29^ Therefore, BRAF inhibitor (BRAFi) monotherapy cannot be recommended as adjuvant treatment for melanoma.

The phase III COMBI-AD trial^30^ included patients with resected AJCC7 stage III (SLN >1 mm) melanoma with *BRAF* V600E or V600K mutations who were randomised to receive either 12 months of adjuvant dabrafenib–trametinib or placebo. At the final analysis (>10 years of follow-up), the median OS was not reached (NR) in either arm (HR 0.80, 95% CI 0.62-1.01, *P* = 0.06). The estimated RFS (HR 0.52, 95% CI 0.43-0.63) and DMFS (HR 0.56, 95% CI 0.44-0.71) both favoured the dabrafenib–trametinib arm.^31^ In a subgroup analysis, patients with a tumour *BRAF* V600E mutation (91%) in particular appeared to derive benefit in terms of OS (HR 0.75, 95% CI 0.58-0.96) and RFS (HR 0.52). The *BRAF* V600K mutation subgroup did not appear to derive any survival benefit (HR 1.95, 95% CI 0.84-4.50), although patient numbers in this group were small and so definitive conclusions cannot be drawn. Translational and retrospective data suggest that patients with advanced-stage melanoma and *BRAF* V600K mutations derive a greater benefit from ICI therapy than BRAF-targeted therapy. Based on these results, adjuvant dabrafenib–trametinib is an SoC adjuvant treatment option for *BRAF* V600E-mutated stage III melanoma and is approved by the EMA.

### Neoadjuvant and neoadjuvant plus adjuvant systemic therapy for resectable stage III melanoma and clinically or radiologically detectable LN metastasis

Prospectively planned treatment with neoadjuvant therapy followed by surgery and adjuvant therapy may also be referred to as perioperative therapy. This approach differs from prospectively planned neoadjuvant therapy alone, where any subsequent systemic therapy may be given depending on the pathological response.

Several early clinical trials investigated neoadjuvant and neoadjuvant plus adjuvant therapy, including ICIs alone or in combination, BRAFi–MEK inhibitor (MEKi) combination therapy and intralesional therapies alone or in combination, with the principal aim of studying the association between pathological response, RFS and OS. In a pooled analysis of data from 633 (77%) clinical trial patients and 185 (23%) real-world patients treated with ICI-based therapy, BRAFi–MEKi targeted therapy or ICI plus targeted therapy, a pathological complete response (pCR) or near pCR occurred in 55% of patients: 51% with targeted therapy, 58% with ICIs and 46% with ICI plus targeted therapy. In patients who achieved a pCR or near pCR, the 3-year RFS rates were 57% with targeted therapy, 93% with ICIs and 85% with ICI plus targeted therapy. In contrast, patients who achieved a pathological partial response (pPR) or pathological non-response (pNR) had 3-year RFS rates of 15% and 13% with targeted therapy, 79% and 41% with ICIs and 88% and 48% with ICI plus targeted therapy, respectively.^32^

The randomised phase II Southwest Oncology Group (SWOG) S1801 trial included patients with histologically confirmed, measurable, clinically detectable and resectable stage IIIB-IV cutaneous, acral and mucosal melanomas without brain metastases (BMs) who were randomised 1 : 1 to receive either adjuvant therapy (upfront surgery followed by 18 doses of pembrolizumab 200 mg q3w) or neoadjuvant plus adjuvant therapy (3 doses of neoadjuvant pembrolizumab followed by surgery and 15 doses of adjuvant pembrolizumab).^33^ The majority of patients [288/313 (92%)] included in this trial had stage III disease. With a median follow-up of 14.7 months, event-free survival (EFS) was significantly longer with neoadjuvant plus adjuvant versus adjuvant therapy (*P* = 0.004 log-rank test); this EFS benefit was consistent across predefined subgroups.^33^ There was no significant difference in OS (HR 0.63, 95% CI 0.32-1.24, one-sided *P* = 0.091), although OS data were immature at the time of reporting.^34^ The AE rates were similar in both groups.^33^ In patients who received neoadjuvant plus adjuvant therapy, 40% achieved a pCR.^35^

NADINA was a phase III, randomised trial of neoadjuvant nivolumab–ipilimumab (two cycles) versus adjuvant nivolumab in 423 patients with biopsy-proven, resectable stage III melanoma involving LNs ± a maximum of three ITMs.^36^ In the neoadjuvant group, only patients who had a pPR or pNR received subsequent adjuvant treatment with either dabrafenib–trametinib (for *BRAF*-mutated melanoma) or nivolumab. The design and dosing schedule for NADINA were based on results from the two neoadjuvant trials that evaluated two cycles of nivolumab–ipilimumab (OpACIN-neo^37^ and PRADO^38^). At a median follow-up of 15.4 months, the estimated 18-month EFS and 18-month DMFS rates were 80.8% in the neoadjuvant group versus 53.9% in the adjuvant group (HR 0.32, 95% CI 0.22-0.48) and 85.7% in the neoadjuvant group versus 62.4% in the adjuvant group (HR 0.37, 95% CI 0.24-0.57), respectively. In the neoadjuvant group, 60.8% of patients had a major pathological response (MPR) defined according to International Neoadjuvant Melanoma Consortium (INMC) criteria (see below). The estimated 18-month RFS and 18-month DMFS rates were 93.1% and 96.9% for patients who had an MPR, 80.5% and 80.5% for those who had a pPR and 55.1% and 60.6% for those who had a pNR, respectively. These findings suggest that for patients who achieve an MPR after two cycles of neoadjuvant nivolumab–ipilimumab, further adjuvant therapy is not needed. However, longer follow-up is required to confirm these results.

PIVOTAL is a randomised phase III trial of neoadjuvant daromun (a combination of two antibody–cytokine fusions L19IL2 and L19TNF) followed by surgery versus upfront surgery in 256 patients with resectable stage III melanoma (60% of patients had cutaneous or subcutaneous metastases and 33% had received prior systemic therapy). At a median follow-up of 21.2 months, RFS [blinded independent central review (BICR) assessment] and DMFS were both significantly longer in the neoadjuvant treatment group (HR 0.59, 95% CI 0.41-0.86, log-rank *P* = 0.005 and HR 0.60, 95% CI 0.37-0.95, *P* = 0.029, respectively). Neoadjuvant therapy also resulted in a pCR rate of 21%. Daromun-related AEs were mostly local events, with limited, low-grade systemic AEs and no autoimmune TRAEs recorded.^39^ Given these data, daromun may be an option for patients with resectable stage III melanoma and cutaneous metastases, but further data are required.

A randomised phase II trial evaluated neoadjuvant talimogene laherparepvec (T-VEC) followed by surgery versus surgery alone in 150 patients with resectable stage IIIB-IV M1a melanoma. At a median follow-up of 32.1 months for the neoadjuvant group and 30.9 months for the surgery group, the 2-year RFS rates were 29.5% and 16.5%, respectively (HR 0.75, 80% CI 0.58-0.96). Neoadjuvant therapy was associated with a pCR rate of 17.1%.^40^

The INMC was established with the aim of developing recommendations for investigating neoadjuvant therapy in melanoma to align future trial designs and correlative analyses.^41^ Although neoadjuvant therapy is not currently approved, it is reimbursed in some countries. Indeed, neoadjuvant therapy may be particularly beneficial in the following clinical situations:

- Patients with resectable stage III melanoma confined to the LNs, detectable by clinical or radiological assessment.
- Patients with resectable ITMs or oligometastatic stage IV disease.

Pathological response of patients who have undergone neoadjuvant treatment should be assessed based on guidance from the INMC (see: https://melanoma-inc.org/). Definitions of best pathological response are provided in Supplementary Table S9, available at https://doi.org/10.1016/j.annonc.2024.11.006.^42^

### Recommendations

#### Treatment of localised melanoma

- Full depth, WLE of primary tumours with safety margins of 0.5 cm for *in situ* melanomas, 1 cm for tumours with a thickness ≤2 mm and 2 cm for tumours >2 mm is recommended [III, B].

#### Treatment of locoregional melanoma

- Patients with pT1b-T4b cN0 cM0 melanoma and a positive SLNB should undergo standard follow-up [III, A] ± systemic therapy according to disease stage (see Figure 1) [I, A].
- CLND is not recommended for patients with a positive SLNB [I, E].
- Patients with a negative SLNB can be offered standard follow-up [III, A], clinical trial participation [V, A] or anti-PD-1 therapy for 12 months (stages IIB-IIC) [I, A].
- Enrolment into a clinical trial wherever possible is preferred [V, A].

#### Treatment of ITMs

- Patients with resectable ITMs should undergo complete excision with clear margins [IV, B]. These patients can also be evaluated for neoadjuvant nivolumab–ipilimumab [I, A; ESMO-Magnitude of Clinical Benefit Scale (ESMO-MCBS) v1.1 score: A; not EMA or FDA approved] followed by adjuvant therapy based on pathological response and *BRAF* status, neoadjuvant plus adjuvant pembrolizumab [II, A; not EMA or FDA approved] or adjuvant therapy [I, A].
- Patients with resectable disease but a short disease-free interval (<6 months), unresectable satellite, ITMs or inoperable primary tumours of the limbs without additional metastases should be treated with systemic therapy [III, B].
- Patients with unresectable satellite or ITMs may be treated with systemic therapy with anti-PD-1 based immunotherapy or BRAFi–MEKi, according to *BRAF* mutation status [I, A].
  ○ Local therapy with T-VEC, [I, B; ESMO-MCBS v1.1 score: 3], isolated limb infusion or isolated limb perfusion [IV, C], RT [IV, C], electrochemotherapy [IV, C] or limited palliative excision [IV, C] can also be considered (no impact on OS).

#### Adjuvant RT

- Adjuvant RT is not routinely recommended [III, D].
- RT can be considered for local tumour control in cases of inadequate resection margins of lentigo maligna [III, B].
- Adjuvant RT to the primary excision site should be considered for patients with desmoplastic or neurotropic melanoma for whom adequate (≥8 mm) pathological resection margins cannot be achieved [IV, C].
- RT could be discussed for patients with an R1 resection (resection with microscopic tumour at the margin) or after resection of bulky LN metastases, especially if further surgical clearance is not feasible [III, C].

#### Adjuvant systemic therapy in stage IIB-IIC melanoma

- Adjuvant therapy with either pembrolizumab [ESMO-MCBS v1.1 score: A] or nivolumab [ESMO-MCBS v1.1 score: A] for 12 months should be considered for patients with stage IIB-IIC disease; treatment discussions with the patient should include consideration of the RFS benefit but lack of mature OS data [I, A].

#### Adjuvant systemic therapy in resected stage III and IV NED

- Adjuvant systemic therapy options are anti-PD-1 therapy (nivolumab for resected stage IIIB-IV NED [I, A; ESMO-MCBS v1.1 score: no evaluable benefit] or pembrolizumab for resected stage III [I, A; ESMO-MCBS v1.1 score: A]) or dabrafenib–trametinib for patients with resected stage III *BRAF* V600E-mutated melanoma [I, A; ESCAT score: I-A].
  ○ For anti-PD-1-based therapy, treatment discussions with the patient should consider the DMFS and RFS benefits but lack of mature OS data compared with placebo [I, A].
  ○ For dabrafenib–trametinib, these discussions should also consider the DMFS and RFS benefits and potential OS benefit for patients with *BRAF* V600E-mutated melanoma [I, A].
  ○ These treatments should be given within 12 weeks of complete resection [I, A].
- Targeted therapy should not be offered to patients with *BRAF* V600K-mutated melanoma in light of the potential detrimental effect on OS reported in the COMBI-AD trial [II, D].
- For patients with AJCC8 stage IIIA and <1 mm tumour burden, adjuvant systemic treatment is generally not recommended [I, D].
- The use of adjuvant nivolumab–ipilimumab is not recommended for resected stage III melanoma [I, D; not EMA or FDA approved].
- Patients with resectable stage IV melanoma can be offered systemic therapy [V, A], clinical trial [V, A] or metastasectomy or local ablative therapy [III, B] followed by adjuvant anti-PD-1 therapy [I, A].
  ○ The use of adjuvant nivolumab–ipilimumab according to the dosing schedule utilised in the phase II IMMUNED trial may be an option for selected patients with resected stage IV melanoma [II, C; not EMA or FDA approved].

### Neoadjuvant and neoadjuvant plus adjuvant systemic therapy in resectable stage III melanoma and clinically or radiologically detectable LN metastasis

- For patients with resectable stage III melanoma and pathologically proven, clinically or radiologically detectable LN metastasis, neoadjuvant nivolumab–ipilimumab [ESMO-MCBS v1.1 score: A; not EMA or FDA approved] followed by surgery should be offered. For patients with an MPR defined according to INMC criteria, adjuvant treatment can be omitted. For patients without an MPR, further treatment should be discussed [I, A].
- Neoadjuvant plus adjuvant pembrolizumab is also recommended for these patients [II, A; not EMA or FDA approved].
- Treatment discussions with the patient regarding neoadjuvant therapy should consider the EFS, DMFS and RFS benefits but lack of mature OS data [I, A].

## Management of Advanced/Metastatic Disease

Some patients with stage IV melanoma present with resectable disease. Although the value of complete surgical resection in such a clinical setting has not been validated in phase III prospective clinical trials, data from phase II trials are available.^43^ Surgery remains an option for selected patients, preferentially combined with adjuvant or neoadjuvant systemic therapies and in a clinical trial setting.

### Treatment of unresectable stage III and IV melanoma

Despite the improvements in OS with currently available systemic treatments (ICIs and targeted therapy), many questions remain unanswered, with resistance still a challenge; therefore, inclusion in clinical trials is a priority in all settings whenever possible.

**Figure 4:**
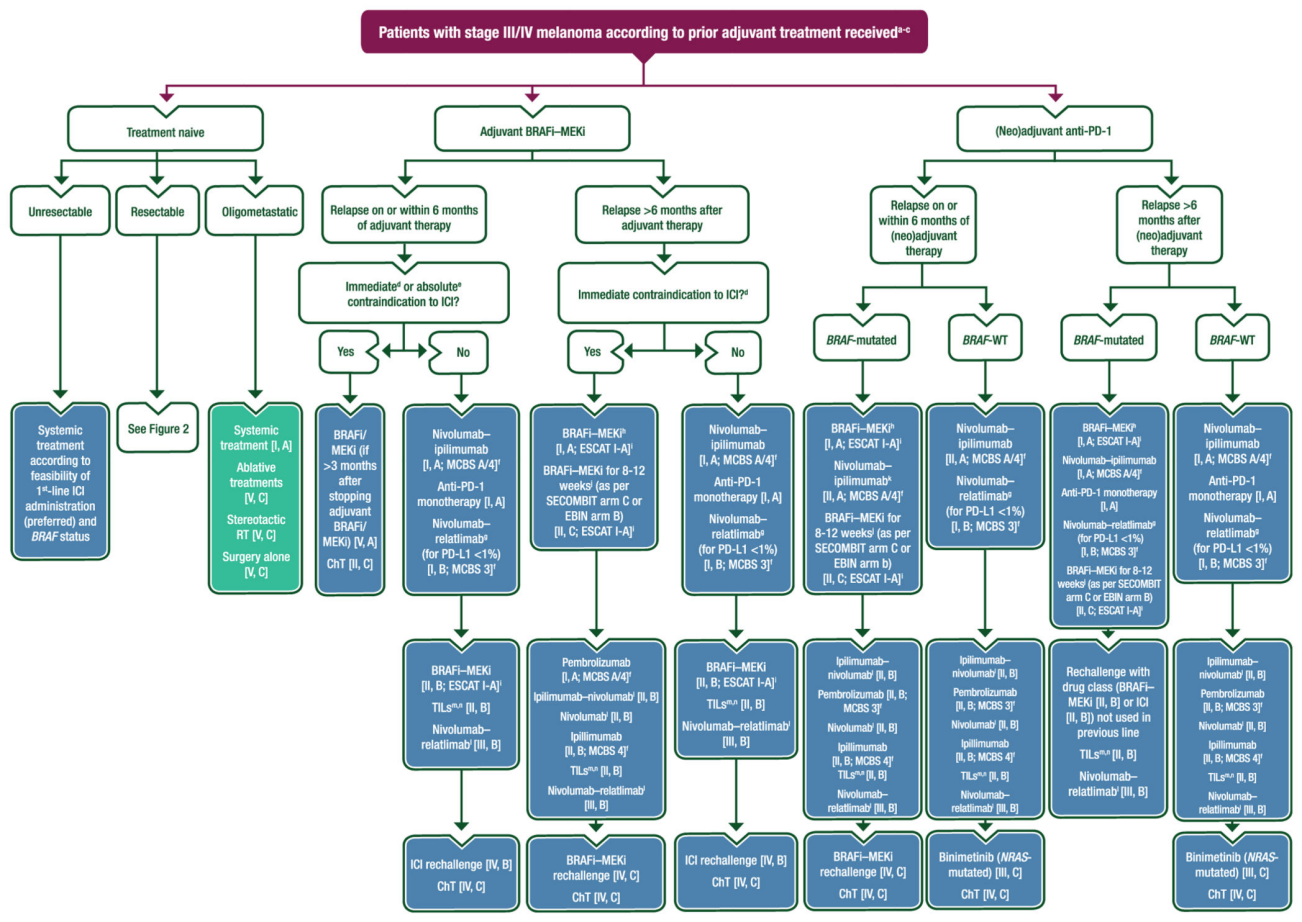
Proposed algorithm for the management of patients with stage III/IV melanoma according to prior adjuvant treatment received. Purple: algorithm title; blue: systemic anticancer therapy; turquoise: combination of treatments and treatment modalities; white: non-treatment aspects. BM, brain metastasis; BRAFi, BRAF inhibitor; ChT, chemotherapy; ctDNA, circulating tumour DNA; EMA, European Medicines Agency; ESCAT, ESMO Scale for Clinical Actionability of molecular Targets; ESMO, European Society for Medical Oncology; FDA, Food and Drug Administration; ICI, immune checkpoint inhibitor; LDH, lactate dehydrogenase; M, metastasis; MCBS, ESMO-Magnitude of Clinical Benefit Scale; MEKi, MEK inhibitor; NGS, next-generation sequencing; PD-1, programmed cell death protein 1; PD-L1, programmed death-ligand 1; PS, performance status; RT, radiotherapy; TIL, tumour-infiltrating lymphocyte; T-VEC, talimogene laherparepvec; WG, working group; WT, wild type. ^a^Patients with metastatic melanoma should have metastases (preferably) or the primary tumour screened for the detection of *BRAF* V600 mutation [IV, A; ESCAT score: I-A]. If no tumour tissue is available, ctDNA may be an alternative [III, C]. ^b^Enrolment into a clinical trial is preferred wherever possible [V, A]. ^c^Additional treatment options include palliative resection [IV, C], RT [IV, B] and/or T-VEC [I, C] for patients with symptomatic extracranial disease; and best supportive and palliative care for all patients [V, A]. Local therapies should also be considered for all patients throughout the disease course, including for resectable recurrence after (neo)adjuvant therapy and, where needed, to achieve local control, with access to tissue for NGS analysis providing the potential for personalised therapy. ^d^Immediate contraindications to ICI include rapid progression, elevated LDH levels, comorbidities and any symptoms that preclude ICI use. In these situations, ICI therapy should be reconsidered as soon as the contraindications are resolved and ICI becomes a viable therapy option. ^e^Absolute contraindications to ICI should be based on a multidisciplinary assessment. ^f^ESMO-MCBS v1.1^78^ was used to calculate scores for therapies/indications approved by the EMA or FDA. The scores have been calculated and validated by the ESMO-MCBS WG and reviewed by the authors (https://www.esmo.org/guidelines/esmo-mcbs/esmo-mcbs-evaluation-forms). ^g^EMA approved for PD-L1 expression <1%, FDA approval is regardless of PD-L1 expression. ^h^For patients in whom the decision to treat with targeted therapy has been made, those who cannot receive a MEKi (e.g. due to cardiovascular comorbidities, a recent BM bleeding event, history of retinal detachment or other ophthalmological contraindications) can be offered encorafenib as monotherapy [II, B; not FDA or EMA approved]. ^i^ESCAT scores apply to genomic alterations only. These scores have been defined by the guideline authors and assisted as needed by the ESMO Translational Research and Precision Medicine Working Group.^77^ ^j^Induction targeted therapy followed by anti-PD-1 therapy is not EMA or FDA approved. The optimal duration of induction targeted therapy is currently unknown. ^k^For patients who do not require a rapid tumour response to therapy due to aggressive disease. ^l^Not EMA or FDA approved for second-line use. ^m^An option for selected young, fit patients with stage IV M1a-c melanoma, PS 0, normal LDH, 1-3 prior treatments and who are able to tolerate TIL-related side-effects. ^n^Not EMA or FDA approved.

Proposed algorithms for the management of unresectable stage III and IV melanoma are provided in Figures 3 and 4.

### First-line treatment

First-line treatment selection depends on the strategy used in the neoadjuvant and/or adjuvant setting as well as the *BRAF* mutational status of the disease. The current first-line SoC treatment options for unresectable stage III/IV melanoma are PD-1 blockade (nivolumab, pembrolizumab), PD-1 blockade combined with CTLA-4 blockade (nivolumab–ipilimumab), PD-1 blockade combined with lymphocyte activation gene-3 (LAG-3) blockade (nivolumab–relatlimab) and, for *BRAF* V600-mutated melanoma, BRAFi (vemurafenib, dabrafenib, encorafenib) combined with MEKi (cobimetinib, trametinib, binimetinib). For unresectable stage IIIB-IIIC or IV M1a disease (AJCC7 criteria), T-VEC is also an option (see ‘Treatment of ITMs’ section above). However, combining T-VEC with PD-1 blockade does not provide any additional clinical benefit. It is worth noting that data for treatments in the unresectable stage III and IV disease setting were generated before these therapies became available in the adjuvant setting for patients with stage II/III melanoma. Therefore, the benefits seen for patients who have received adjuvant therapy may be different from the data reported here. Prospective data regarding the optimal treatment strategy for patients with unresectable stage III or IV melanoma who have received prior adjuvant therapy are required.

#### Immunotherapy

The superiority of nivolumab over dacarbazine (DTIC) chemotherapy (ChT) for the first-line treatment of patients with *BRAF*-wild-type (WT) melanoma was demonstrated in the prospective randomised CheckMate 066 trial, with an HR for death of 0.42 (99.79% CI 0.25-0.73, *P* < 0.001) and an HR for death or progression of disease of 0.43 (95% CI 0.34-0.56, *P* < 0.001).^44^ Superiority of PD-1 blockade (nivolumab, pembrolizumab) over ipilimumab was demonstrated in two prospective randomised trials, CheckMate 067 and KEYNOTE-006.^45,46^ After a minimum follow-up of 10 years, CheckMate 067 had an HR for death for nivolumab versus ipilimumab of 0.63 (95% CI 0.52-0.76)^47^ and KEYNOTE-006 (patients included in the KEYNOTE-587 extension study only) had an HR for death for pembrolizumab (both dose arms combined) versus ipilimumab of 0.71 (95% CI 0.60-0.85).^48^ Based on these trials, PD-1 blockade is now an SoC option for all patients, regardless of tumour *BRAF* status, in the first-line setting.

The benefit of adding ipilimumab to nivolumab was also assessed in the CheckMate 067 trial. Treatment with nivolumab–ipilimumab according to this trial’s dosing regimen resulted in numerically higher response rates (RRs) and longer response durations, time to subsequent therapies, patients alive after stopping therapy, progression-free survival (PFS) and OS.^45^ By study design, the two nivolumab-containing arms could not be compared. At a minimum follow-up of 10 years, the median OS was 71.9, 36.9 and 19.9 months in the nivolumab–ipilimumab, nivolumab-only and ipilimumab-only groups, respectively. Median MSS was NR, 49.4 and 21.9 months, respectively, and median duration of response (DoR) was NR (>120 months), 103.2 months and 19.2 months, respectively.^47^ Grade 3-4 AEs were reported in 55.0%, 16.3% and 27.3% of patients in the nivolumab–ipilimumab, nivolumab-only and ipilimumab-only groups, respectively,^49^ with no new safety signals observed in subsequent trial reports.^50^

A phase IIIb/IV trial showed that an alternative dosing schedule of nivolumab–ipilimumab (nivolumab 3 mg/kg plus ipilimumab 1 mg/kg) was associated with reduced toxicity.^51^ Therefore, this dosing schedule could be discussed for some frail patients. However, as this trial was designed to evaluate the safety (and not the efficacy) profile of this alternative dosing schedule, its general use cannot be recommended.

More recently, an improvement in PFS has been reported for relatlimab–nivolumab. In the phase II/III, multicentre, double-blind, randomised RELATIVITY-047 trial, relatlimab–nivolumab was administered as a fixed-dose q4w to patients with previously untreated unresectable or metastatic melanoma. A median PFS of 10.1 months was reached with relatlimab–nivolumab versus 4.6 months with nivolumab (HR 0.75, 95% CI 0.62-0.92, *P* = 0.006 log-rank test). The 12-month PFS rate was 47.7% with relatlimab–nivolumab versus 36% with nivolumab. PFS across key subgroups also favoured relatlimab–nivolumab over nivolumab. Grade 3/4 TRAEs occurred in 18.9% of patients in the relatlimab–nivolumab group versus 9.7% in the nivolumab group.^52^ In an updated analysis at a median follow-up of 19.3 months, the HR for PFS by BICR in patients with a PD-L1 expression of <1% (*n* = 209) and ≥1% (*n* = 147) was 0.68 (95% CI 0.53-0.86) and 0.96 (95% CI 0.70-1.31), respectively. The OS HRs in these subsets were 0.78 (95% CI 0.59-1.04) and 0.84 (95% CI 0.57-1.24), respectively.^53^ Based on these data, relatlimab–nivolumab can be considered as a first-line treatment option. For patients who need to discontinue relatlimab–nivolumab due to toxicity, continuation of anti-PD-1 monotherapy can be discussed. In July 2022, the EMA approved the use of relatlimab–nivolumab for the first-line treatment of adults and adolescents (≥12 years of age) with advanced melanoma and a PD-L1 tumour expression of <1%.

Pembrolizumab–lenvatinib as first-line treatment for patients with unresectable or metastatic melanoma was evaluated in the phase III LEAP-003 trial. Despite a significant improvement in PFS observed for the combination in an early interim analysis, findings from a subsequent analysis showed no OS benefit and a significant increase in toxicity and the trial was subsequently discontinued.^54^

Given this collective evidence, treatment decisions should be tailored based on several parameters, including prior (neo)adjuvant therapy received, timing of recurrence on/after adjuvant therapy, resectability status, suitability to receive ICI therapy and PD-L1 status. For patients eligible to receive ICI therapy, the treatment choice of single-agent PD-1 blockade versus nivolumab–ipilimumab or relatlimab–nivolumab should be individualised to each patient.

#### Targeted therapy

In case of *BRAF*-mutated melanoma, additional first-line options are provided by BRAFis and MEKis. Combined BRAFi–MEKi is superior to single-agent BRAFi in terms of RRs, PFS and OS.^55–58^ However, findings from a *post hoc* analysis suggest that encorafenib as monotherapy provides a similar OS benefit to combined BRAFi–MEKi (HR 0.93, 95% CI 0.73-1.18) and so could be an option for patients with contraindications to MEKis, although encorafenib is not EMA or FDA approved as monotherapy.^59^ In addition to improved efficacy, skin-related side-effects and the incidence of squamous-cell carcinomas are reduced with the combination, although MEKis add specific toxicities (e.g. muscle, heart, eyes). Single-agent BRAFis should only be used in case of an absolute contraindication for MEKis.

#### Treatment selection

First-line treatment decisions between targeted therapies and immunotherapies have been evaluated in several prospective trials, with the aim of defining the best sequencing approach. The phase III DREAMSEQ trial included 265 patients with treatment-naive *BRAF* V600-mutated metastatic melanoma, stratified by Eastern Cooperative Oncology Group PS 0 or 1 and lactate dehydrogenase (LDH) level; patients with untreated melanoma BMs (MBMs) were excluded. Patients were randomised 1 : 1 to receive either nivolumab–ipilimumab (arm A) or dabrafenib–trametinib (arm B), and at disease progression, patients in arm A received dabrafenib–trametinib (arm C) and patients in arm B received nivolumab–ipilimumab (arm D). The median DoR was significantly longer for arm A than for arm B (NR versus 12.7 months, *P* < 0.001). The PFS showed a trend in favour of arm A (log-rank *P* = 0.054). The 2-year OS rate for those starting in arm A was 71.8% and was 51.5% for those starting with arm B (log-rank *P* = 0.010). It is worth noting that only ~50% of patients who had disease progression in arm A or B were enrolled into arm C or D, respectively, since most died within 6 months of their initial disease progression, many due to MBMs. Also, given the significant difference in terms of 2-year OS rates between arms A and B, the data safety monitoring committee recommended the study be closed to accrual and for patients in arm B to be given the option to switch to arm D without disease progression. Given this, the initial question regarding the best therapeutic sequence was not completely answered. Overall, grade ≥3 toxicity was 60% in arm A and 52% in arm B. Grade 5 TRAEs included two patients in arm A and one in arm C.^60^

In the randomised, three-arm, non-comparative phase II SECOMBIT trial, patients with previously untreated, metastatic *BRAF* V600-mutated melanoma were randomly assigned to arm A (*n* = 69; encorafenib–binimetinib until disease progression and then ipilimumab–nivolumab), arm B (*n* = 71; ipilimumab–nivolumab until disease progression and then encorafenib–binimetinib) or arm C (*n* = 69; encorafenib–binimetinib for 8 weeks followed by ipilimumab–nivolumab until disease progression and then encorafenib–binimetinib). At a median follow-up of 32.2 months, median OS was NR in any arm and >30 patients were alive in all arms. No new safety signals emerged.^61^ The 5-year OS rates were 45% in arm A, 52% in arm B and 57% in arm C.^62^

The randomised, phase II EBIN trial evaluated a total of 2 years of therapy, either with immunotherapy (nivolumab–ipilimumab for four cycles followed by nivolumab; arm A) or encorafenib–binimetinib for 3 months followed by immunotherapy, as per arm A (arm B) in 271 patients with *BRAF*-V600E/K unresectable stage III/IV melanoma. At a median follow-up of 21 months, there was no PFS benefit associated with the addition of induction targeted therapy (HR 0.87, 90% CI 0.67-1.12, *P* = 0.36). However, findings from a prespecified subgroup analysis suggested a PFS benefit for induction targeted therapy among patients with LDH >2× the upper limit of normal (ULN; HR 0.46, 95% CI 0.21-1.03). In a *post hoc* analysis, patients with liver metastases also benefited from the sequential design (HR 0.48, 95% CI 0.28-0.80).^63^

Based on these results, first-line nivolumab–ipilimumab is the preferred treatment when this can be safely delivered for the first few months (i.e. when a rapid response is not required due to aggressive/symptomatic disease), with targeted therapies reserved for subsequent treatment lines. The optimal duration of induction targeted therapy and the best targeted therapy combination is currently unknown.

#### Oligometastatic disease

Oligometastatic disease is difficult to define. It is dependent on the number and localisation of metastatic sites and can be resectable or unresectable, but there is currently no consensus regarding its definition. Treatment options mimic those for patients with resectable stage IV melanoma and include systemic therapy, surgery, local treatment (stereotactic RT or ablative therapy) or a clinical trial (see Figure 4). However, based on results from trials evaluating neoadjuvant systemic therapy, the use of first-line systemic therapy instead of ablative therapy, even in patients with resectable oligometastatic disease, seems preferable and should be discussed. Data from clinical trials investigating this question are required.

### Second-line treatment

Second-line treatment selection depends on the strategy used in the neoadjuvant, adjuvant and first-line metastatic setting as well as the *BRAF* mutational status of the disease, as illustrated in Figure 4. Clinical trials should always be the first choice, when available, based on an appropriate backbone regimen and comparator arm if randomised. At all time points for patients with oligometastatic disease, ablative therapy can be considered.

#### *BRAF*-WT melanoma

For *BRAF*-WT melanoma, approved second-line options are very limited. Thus, consideration for clinical trials and/or personalised approaches is appropriate. If the first-line treatment was anti-PD-1 monotherapy or if patients had primary refractory disease following anti-PD-1 therapy, ipilimumab and ipilimumab–nivolumab are options based on results from the phase II SWOG S1616 trial.^64–66^ In this trial, treatment with ipilimumab–nivolumab was associated with a statistically significant improvement in PFS compared with ipilimumab alone (HR 0.63, 90% CI 0.41-0.97, one-sided *P* = 0.04).^66^ Nivolumab–relatlimab might also represent an option after failure of single-agent anti-PD-1 therapy; in the phase I/IIa RELATIVITY-020 trial, objective response rates (ORRs) of 12.0% and 9.2% were reported for patients who had progressed after one or more than one prior anti-PD-1-containing regimen, respectively. Corresponding median PFS values were 2.1 and 3.2 months, respectively.^67^

In some cases, such as (i) toxicity to anti-PD-1-based immunotherapy which precludes the use of second-line anti-PD-1-based therapy, (ii) rapidly progressing disease or (iii) high tumour volume with symptomatic disease, clinical trials including bispecifics, T-cell engagers, etc. should be a preferred option. ChT with DTIC, carboplatin–paclitaxel, temozolomide or fotemustine can be discussed. However, none of these ChT regimens provide an OS advantage^68^ and they are associated with a low RR, short PFS and increased toxicity in patients who have progressed on ICI therapy.^69^

TIL therapy is another option which can be manufactured using different techniques. However, one of the limitations of this therapy is the time needed for manufacturing, which is currently 3-6 weeks, making it an option only for a selected group of patients. TIL therapy is currently not EMA approved for use in this setting.

In a phase II trial of TIL therapy, lifileucel (an autologous, centrally manufactured TIL product) demonstrated durable responses in patients with previously treated metastatic melanoma and limited treatment options. In this trial, patients received a non-myeloablative lymphodepletion regimen, a single infusion of lifileucel and up to six doses of high-dose interleukin-2 (IL-2). The ORR was 36%, with two complete responses (CRs) and 22 partial responses. The disease control rate (DCR) was 80% and the median DoR was NR after a median follow-up of 18.7 months. In the subset of patients with primary refractory disease following prior anti-PD-1 therapy, the ORR and DCR were 41% and 81%, respectively. The safety profile was consistent with AEs associated with non-myeloablative lymphodepletion and IL-2.^70^

In an open-label phase III trial, 168 patients (86% with anti-PD-1-refractory disease) with unresectable stage IIIC-IV melanoma (AJCC7 criteria) were randomised 1 : 1 to receive either TILs (manufactured at each trial centre) or ipilimumab. Infusion of TILs was preceded by non-myeloablative, lymphodepleting ChT followed by high-dose IL-2. After a median follow-up of 33.0 months, median PFS was 7.2 months for the TIL group versus 3.1 months for the ipilimumab group (HR 0.50, 95% CI 0.35-0.72, *P* < 0.001). The ORR was 49% for the TIL group and 21% for the ipilimumab group, with 20% and 7% achieving a CR, respectively. Median OS was 25.8 months for the TIL group and 18.9 months for the ipilimumab group (HR 0.83, 95% CI 0.54-1.27, *P* = 0.39). Grade ≥3 TRAEs occurred in all TIL-treated patients and 57% of ipilimumab-treated patients.^71^ Currently, TIL therapy remains a highly toxic treatment option for selected patients who can tolerate its side-effects, administered within regional reference centres.^72^ Current clinical trial evidence suggests that those who derive most benefit are young patients with stage IV M1a-c melanoma, PS 0, normal LDH and one to three prior treatments.^73^

In the phase II, LEAP-004 trial, pembrolizumab–lenvatinib was evaluated in patients progressing within 12 weeks of the last dose of an anti-PD-1 inhibitor, given as monotherapy or with other therapies, including CTLA-4 inhibitors. In the overall population, after a median follow-up of 15.3 months, the ORR was 21.4%, median DoR was 8.3 months, median PFS was 4.2 months and median OS was 14.0 months.^74^ Pembrolizumab–lenvatinib is not EMA or FDA approved for use in this setting.

#### *BRAF*-mutated melanoma

For *BRAF*-mutated melanoma, all the options available for *BRAF*-WT melanoma are still valid, with the addition of combined BRAFi–MEKi therapy if not already used as the immediate prior treatment. BRAFi–MEKi therapy after disease progression with first-line immunotherapy should be offered.

#### *NRAS*-mutated melanoma

For *NRAS*-mutated melanoma, due to the limited efficacy of MEKis, first-line immunotherapy options are identical to those for *NRAS*-WT melanoma. Binimetinib as a single agent, however, can be considered for patients who do not benefit from prior anti-PD-1 therapy, in accordance with findings from the NEMO trial, but there is no OS benefit and its use as monotherapy is not EMA approved.^75^

### Subsequent lines

Subsequent lines of therapy are not currently evidence based. Clinical trials or rechallenge, either with targeted therapy (for patients with *BRAF*-mutated melanoma) or immunotherapies, can be an option^76^ (see Figure 4).

### MBMs

**Figure 5:**
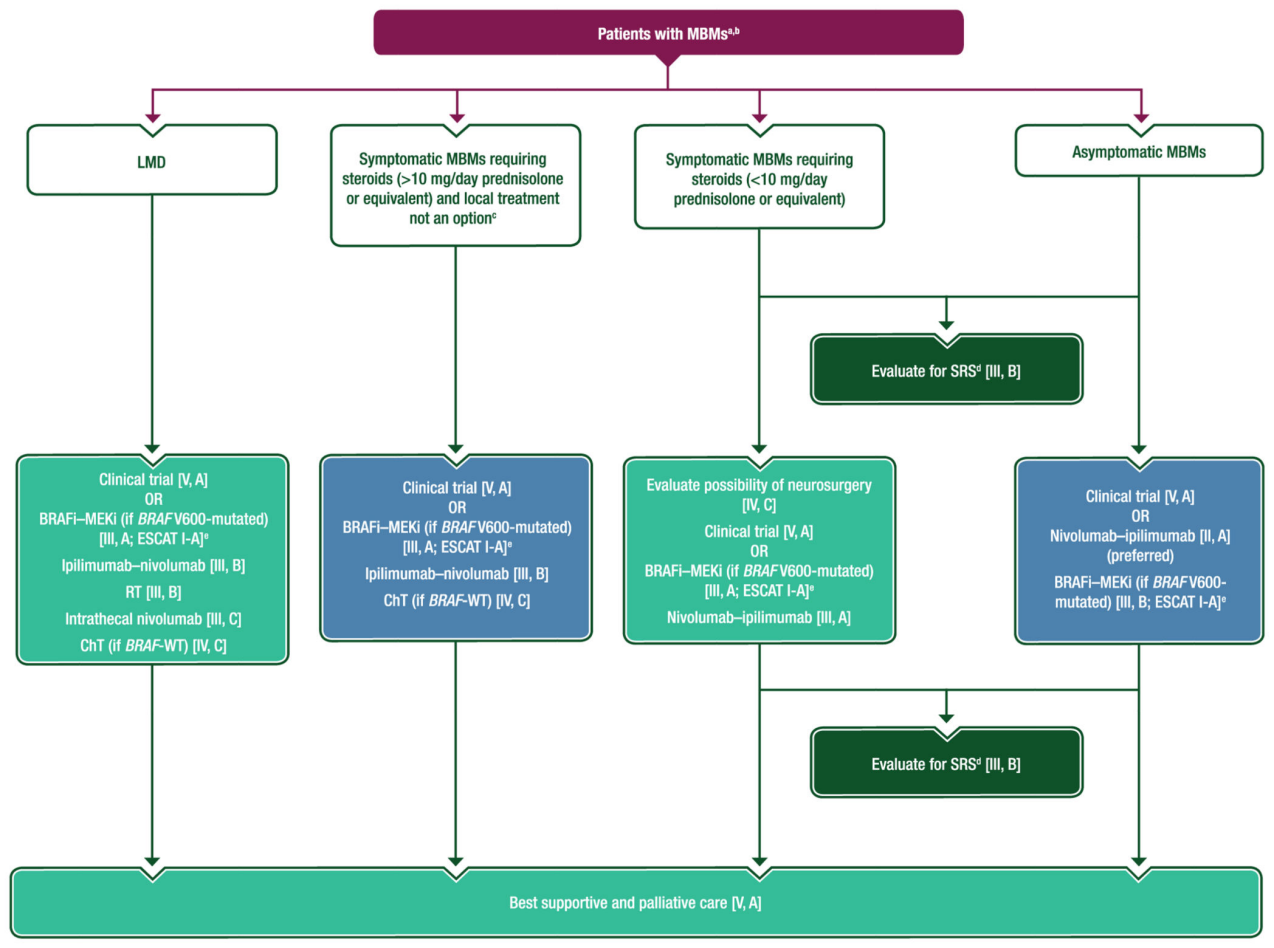
Proposed algorithm for the management of patients with MBMs. Purple: algorithm title; blue: systemic anticancer therapy; turquoise: combination of treatments and treatment modalities; dark green, RT; white: non-treatment aspects. BRAFi, BRAF inhibitor; ChT, chemotherapy; EMA, European Medicines Agency; ESCAT, ESMO Scale for Clinical Actionability of Molecular Targets; ESMO, European Society for Medical Oncology; FDA, Food and Drug Administration; LMD, leptomeningeal disease; MBM, melanoma brain metastasis; MEKi, MEK inhibitor; MRI, magnetic resonance imaging; RT, radiotherapy; SRS, stereotactic radiosurgery; WT, wild-type. ^a^Enrolment into a clinical trial wherever possible is preferred [V, A]. ^b^None of the systemic treatment options listed are EMA or FDA approved to treat MBMs. ^c^In patients where local treatment has been discounted due to the number and/or volume of MBMs, evaluate for the possibility of resection of dominant lesion(s). ^d^Early concurrent SRS may be preferred over late SRS as salvage treatment [IV, C]. Close monitoring with MRI is recommended so that SRS can be added when indicated [IV, B]. ^e^ESCAT scores apply to genomic alterations only. These scores have been defined by the guideline authors and assisted as needed by the ESMO Translational Research and Precision Medicine Working Group.^77^

A proposed algorithm for the management of patients with MBMs is provided in Figure 5. Details of management are provided in Supplementary Material Section 6, available at https://doi.org/10.1016/j.annonc.2024.11.006.

### Predictive and prognostic biomarkers

Information regarding potential predictive and prognostic markers in melanoma is provided in Supplementary Material Section 6, available at https://doi.org/10.1016/j.annonc.2024.11.006.

### Recommendations

#### General recommendations

- Patients with metastatic melanoma should have metastases (preferably) or the primary tumour screened for the detection of *BRAF* V600 mutation [IV, A; ESCAT score: I-A].
  ○ If no tumour tissue is available, circulating tumour DNA may be an alternative [III, C].
  Enrolment into a clinical trial wherever possible is preferred [V, A].
- In addition to the treatment options outlined below, palliative resection [IV, C] and/or RT [IV, B] and/or T-VEC [I, C] can be considered for symptomatic extracranial disease.

#### First-line treatment

- First-line treatment decisions must take into consideration prior neoadjuvant and/or adjuvant therapy received, timing of recurrence on/after adjuvant therapy, resectability status and suitability to receive ICI therapy, as outlined in Figure 4 [V, A]. Primary or secondary resistance must be considered as this is also an eligibility criterion for clinical trials in pretreated patients [V, A].
- Patients with treatment-naive resectable disease can be offered the following:
  ○ Stage III:
    ▪ Wide excision of the primary tumour [III, B].
    ▪ Neoadjuvant nivolumab–ipilimumab [I, A; ESMO-MCBS v1.1 score: A; not EMA or FDA approved] followed by adjuvant therapy based on pathological response and *BRAF* status.
    ▪ Neoadjuvant plus adjuvant pembrolizumab [II, A; not EMA or FDA approved].
    ▪ Adjuvant anti-PD-1 therapy [I, A] or dabrafenib–trametinib for *BRAF* V600E-mutated tumours [I, A].
  ○ Stage IV:
    ▪ Clinical trial [V, A].
    ▪ Metastasectomy or local ablative therapy [III, B] followed by adjuvant anti-PD-1 therapy [I, A].
    ▪ Anti-PD-1 therapy alone [V, A].
- For patients with unresectable disease, first-line ipilimumab–nivolumab [ESMO-MCBS v1.1 score: A/4] is a preferred option for all patients regardless of *BRAF* status when this can be safely delivered for the first few months (i.e. when a rapid response is not required due to aggressive/symptomatic disease) [I, A].
- First-line nivolumab [I, A; ESMO-MCBS v1.1 score: A/4] or pembrolizumab [I, A; ESMO-MCBS v1.1 score: A/4] is also recommended.
  ○ Nivolumab–relatlimab can be offered as first-line treatment but EMA approval is only for patients with tumour cell PD-L1 expression <1% [I, B; ESMO-MCBS v1.1 score: 3; EMA approved for PD-L1 expression <1%, FDA approval is regardless of PD-L1 expression].
- If anti-PD-1-based therapy is not available or patients are considered ineligible for its use, BRAFi–MEKi combination therapy (dabrafenib–trametinib [ESMO-MCBS v1.1 score: 5]; vemurafenib–cobimetinib [ESMO-MCBS v1.1 score: A/5]; binimetinib–encorafenib [ESMO-MCBS v1.1 score: A/5]) is also an option in the first line for patients with *BRAF*-mutated melanoma [I; A; ESCAT score: I-A].
  ○ BRAFi–MEKi for 8-12 weeks followed by ipilimumab–nivolumab (as per SECOMBIT arm C or EBIN arm B) is also an option, especially for patients with high LDH levels and/or liver metastases [II, C; ESCAT score: I-A; induction targeted therapy is not EMA or FDA approved].
  ○ For patients in whom the decision to treat with targeted therapy has been made, those who cannot receive a MEKi (e.g. due to cardiovascular comorbidities, a recent BM bleeding event, history of retinal detachment or other ophthalmological contraindications) can be offered encorafenib as monotherapy [II, B; not FDA or EMA approved].
- Patients with *BRAF*-mutated melanoma who have relapsed on or within 6 months of adjuvant BRAFi–MEKi therapy and who have an immediate or absolute contraindication to ICI can be offered the following first-line treatments:
  ○ BRAFi/MEKi (if >3 months after stopping adjuvant BRAFi/MEKi) [V, A].
  ○ ChT [II, C] (no OS benefit).
- First-line immunotherapy options for patients with *NRAS*-mutated melanoma are identical to those for patients with *NRAS*-WT disease [I, A].

### Oligometastatic disease

- Treatment options for patients with oligometastatic disease mimic those for patients with resectable stage IV melanoma and include systemic therapy [I, A], surgery [V, C], local treatment (stereotactic RT or ablative therapy [V, C]) or a clinical trial [V, A].

#### Second-line treatment

- Treatment options for the second-line setting depend on the therapy used in the first line and include ipilimumab–nivolumab [II, B], pembrolizumab [I, A; ESMO-MCBS v1.1 score: A/4], nivolumab [II, B], ipilimumab [II, B; ESMO-MCBS v1.1 score: 4] and BRAFi–MEKi combination therapy for patients with *BRAF*-mutated melanoma [II, B; ESCAT score: I-A]. Only pembrolizumab and ipilimumab monotherapy are EMA or FDA approved for second-line use.
- Nivolumab–relatlimab might also represent an option after failure of single-agent anti-PD-1 therapy [III, B; not EMA or FDA approved as second-line therapy].
- TIL therapy is an aggressive treatment option for selected patients (young, stage IV M1a-c melanoma, PS ≤1, LDH <2× ULN and 1-3 prior treatments) who can tolerate its side-effects [II, B; not EMA or FDA approved].

#### Subsequent lines of treatment

- Third-line treatment rechallenge with the drug class (BRAFi–MEKi [IV, C] or ICI [IV, B]) not used in the immediate previous line can be considered, if feasible.
- If clinical trials, ICIs or BRAFis/MEKis are not available, ChT may be administered as later-line therapy [IV, C], with modest activity and no impact on OS.
- For patients with *NRAS*-mutated melanoma, binimetinib as a single agent can be offered to patients who do not benefit from prior anti-PD-1 therapy [III, C].

#### MBMs

- There are currently no systemic treatment options specifically approved for use in treating MBMs; enrolment into a clinical trial wherever possible is preferred [V, A].
- Patients with MBMs should be evaluated for stereotactic radiosurgery (SRS) [III, B]. Early concurrent SRS may be preferred over late SRS as salvage treatment [IV, C]. Since multiple sessions of SRS can be carried out at different time points of the disease course, close monitoring using MRI is recommended so that SRS can be added when indicated [IV, B].
- Patients with asymptomatic MBMs should preferably be treated upfront with nivolumab–ipilimumab [II, A].
  ○ If unsuitable for immunotherapy, patients with asymptomatic MBMs and *BRAF* V600-mutated melanoma can be offered BRAFi–MEKi [III, B; ESCAT I-A].
- For patients with symptomatic MBMs requiring steroids (<10 mg/day prednisolone or equivalent):
  ○ BRAFi–MEKi if *BRAF* V600-mutated can be offered [III, A; ESCAT I-A].
  ○ Nivolumab–ipilimumab (*BRAF*-mutated or *BRAF*-WT) can be offered [III, A].
  ○ Neurosurgery should be discussed, especially if an accessible, resectable tumour is causing symptoms as this may render the patient asymptomatic and provide a bridging strategy to nivolumab–ipilimumab [IV, C].
- Patients with MBMs and neurological symptoms requiring steroids (>10 mg prednisolone/day or equivalent) for whom local therapy is not an option can be considered for the following:
  ○ Clinical trial [V, A].
  ○ BRAFi–MEKi (if *BRAF* V600-mutated) [III, A; ESCAT I-A].
  ○ Ipilimumab–nivolumab [III, B].
  ○ ChT (if *BRAF*-WT) [IV, C].
- Patients with leptomeningeal disease can also receive the above therapies. Local treatment (RT [III, B] or intrathecal nivolumab [III, C]) can also be considered.
- Best supportive and palliative care should be discussed and activated for all patients with MBMs [V, A].

## Self-Examination, Risk Assessment and Follow-Up

**Figure 6:**
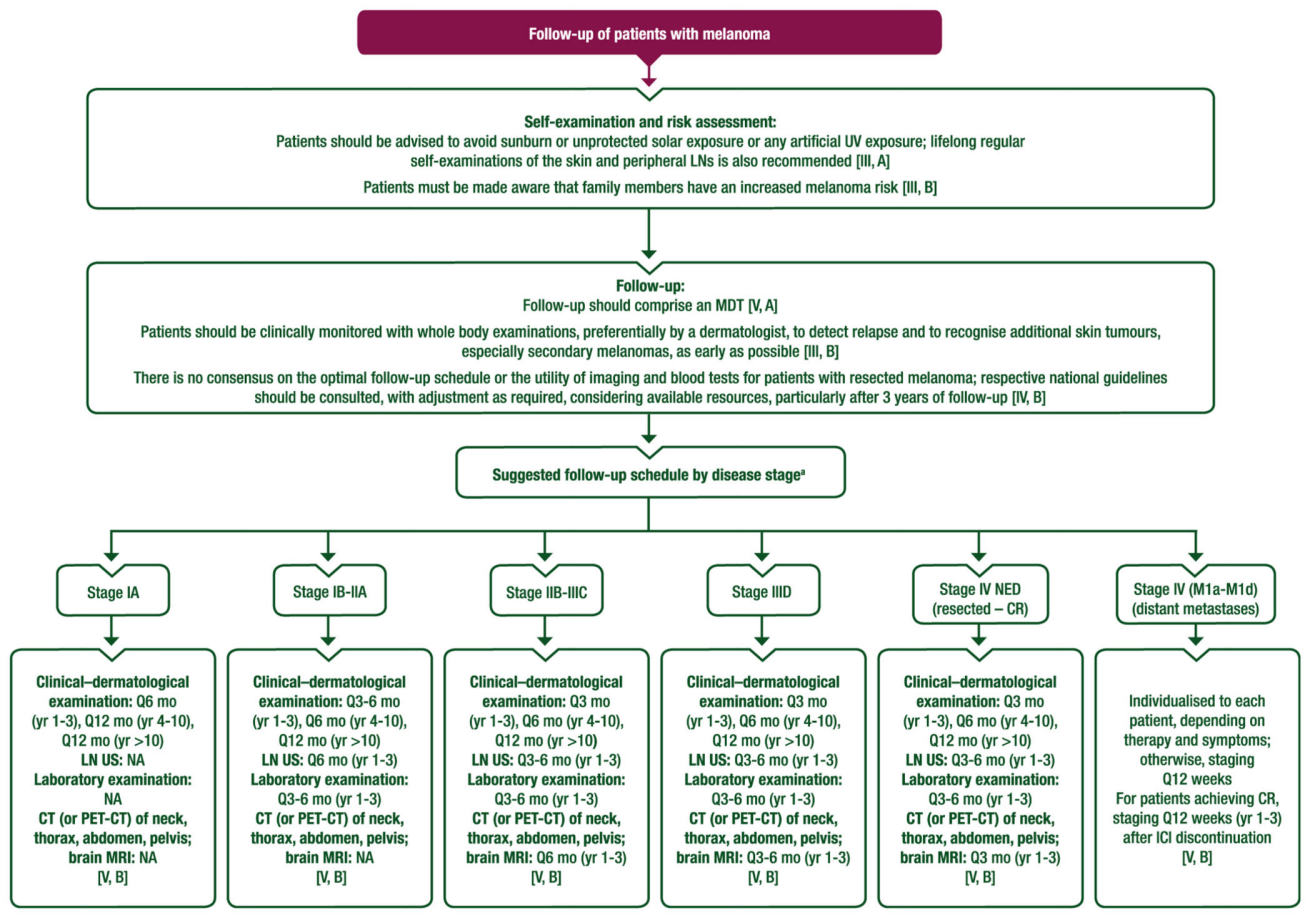
Proposed algorithm for the follow-up of patients with melanoma. Purple: algorithm title; white: non-treatment aspects. CR, complete response; CT, computed tomography; ICI, immune checkpoint inhibitor; LN, lymph node; MDT, multidisciplinary team; mo, month; MRI, magnetic resonance imaging; NA, not applicable; NED, no evidence of disease; PET, positron emission tomography; Q, every; US, ultrasound; UV, ultraviolet; yr, year. ^a^The follow-up schedule should be tailored to each individual patient, considering the disease stage, individual risk and personal needs of the patient, and may include clinical–dermatological examination, LN US, laboratory examinations and imaging [V, B].

A proposed algorithm for the follow-up of patients with melanoma is provided in Figure 6. Details of follow-up are provided in Supplementary Material Section 7, available at https://doi.org/10.1016/j.annonc.2024.11.006.

### Recommendations

- Patients with melanoma should be advised to avoid sunburn or unprotected solar exposure or any artificial UV exposure; lifelong regular self-examinations of the skin and peripheral LNs is also recommended [III, A].
- Patients must be made aware that family members have an increased melanoma risk [III, B].
- Follow-up should comprise a multidisciplinary approach, including oncologists, dermatologists and other specialties, as required by each individual patient’s prior therapy and needs [V, A].
- During melanoma follow-up, patients should be clinically monitored with whole-body examinations, preferentially by a dermatologist, to detect relapse and to recognise additional skin tumours, especially secondary melanomas, as early as possible [III, B].
- There is no consensus on the optimal follow-up schedule or the utility of imaging and blood tests for patients with resected melanoma; respective national guidelines should be consulted, with adjustment as required, considering available resources, particularly after 3 years of follow-up [IV, B].
- The follow-up schedule should be tailored to each individual patient, considering the disease stage, individual risk and personal needs of the patient, and may include clinical–dermatological examination, LN US, laboratory examinations and imaging, as outlined in Figure 6 [V, B].

## Methodology

This CPG was developed in accordance with the ESMO standard operating procedures for CPG development (https://www.esmo.org/Guidelines/ESMO-Guidelines-Methodology). All recommendations provided are based on current scientific evidence and the authors’ collective expert opinion. Where recommendations for multiple different treatment options exist, prioritisation is illustrated by ordering these options according to: level of evidence (LoE) and grade of recommendation (GoR); where equal, by ESMO-MSBC score; where equal, by alphabetical order. The relevant literature has been selected by the expert authors. A table of ESCAT scores is included in Supplementary Table S5, available at https://doi.org/10.1016/j.annonc.2024.11.006. ESCAT scores have been defined by the authors, assisted if needed by the ESMO Translational Research and Precision Medicine Working Group (WG).^77^ A table of ESMO-MCBS scores is included in Supplementary Table S10, available at https://doi.org/10.1016/j.annonc.2024.11.006. ESMO-MCBS v1.1^78^ was used to calculate scores for new therapies/indications approved by the EMA or FDA (https://www.esmo.org/Guidelines/ESMO-MCBS). The scores have been calculated and validated by the ESMO-MCBS WG and reviewed by the authors. The FDA/EMA or other regulatory body approval status of new therapies/indications is reported at the time of writing this CPG. LoEs and GoRs have been applied using the system shown in Supplementary Table S11, available at https://doi.org/10.1016/j.annonc.2024.11.006.^79^ Statements without grading were considered justified standard clinical practice by the authors. For future updates to this CPG, including eUpdates and Living Guidelines, please see the ESMO Guidelines website: https://www.esmo.org/guidelines/guidelines-by-topic/melanoma.

## Supplementary Material

Supplementary Materials
