# Supplementary material for: Cutaneous melanoma: ESMO Clinical Practice Guideline for diagnosis, treatment and follow-up
Source: Ann Oncol. Author manuscript; Available in PMC 2026 Jan 16. (PMC7618628; doi:10.1016/j.annonc.2024.11.006)
Supplement: Supplementary Materials [file EMS212049-supplement-Supplementary_Materials.pdf]

Cutaneous melanoma: ESMO Clinical Practice Guideline for diagnosis, treatment and follow-up

SUPPLEMENTARY MATERIAL

SECTION 1 – MUCOSAL MELANOMA

Mucosal melanoma is a rare and aggressive subtype of melanoma that is associated with a particularly poor prognosis.[1] Its genetic profile differs from that of ultraviolet (UV)-induced melanomas, with a lower mutational burden and more structural variants. Compared with cutaneous melanoma, mucosal melanoma is associated with a lower frequency of *BRAF* (0%-21% versus 42%-50%) and *RAS* mutations (5%-25% versus 30%) but a higher frequency of *KIT* mutations (10%-15% versus 5%-10%).[2] Given the rarity of mucosal melanoma, there is limited evidence to support the optimal treatment approach, and in clinical practice, the management of these patients closely mimics that for cutaneous melanoma. Moreover, patients with mucosal melanoma have been excluded from the majority of trials evaluating systemic therapy in melanoma. Where included, patient numbers are low and the effectiveness of either immunotherapy or targeted therapy is mostly limited (See **Supplementary Table S1**, available at *Annals of Oncology* online). Possible exceptions include the phase II/III RELATIVITY-047 trial of relatlimab–nivolumab versus nivolumab in previously untreated, unresectable or metastatic melanoma, where relatlimab–nivolumab was associated with a progression-free survival (PFS) benefit over nivolumab in patients with mucosal melanoma [n = 51, 23 treated with relatlimab–nivolumab; hazard ratio (HR) 0.64, 95% confidence interval (CI) 0.32-1.25] (overall study population N = 714, PFS HR 0.78, 95% CI 0.64-0.94).[3] In the phase II C-144-01 trial of lifileucel [an autologous, centrally manufactured tumour-infiltrating lymphocyte (TIL) product] in patients with advanced melanoma who had progressed after anti-programmed cell death protein 1 (PD-1)-based therapy, lifileucel was associated with a promising objective response rate (ORR) of 50% in 12 patients with mucosal melanoma[4] (overall study population N = 153, ORR 31.4%[5]). A pooled analysis of data from five clinical trials (three phase III and two phase I trials) suggested an improved PFS and ORR with nivolumab–ipilimumab(n = 35) versus nivolumab monotherapy (n = 86) or ipilimumab monotherapy (n = 36) in patients with mucosal melanoma (median PFS 5.9 months, 3.0 months and 2.7 months; ORR 37.1%, 23.3% and 8.3%, respectively).[6] Thus, although

some therapies may hold promise for the management of mucosal melanoma, further research is required to improve outcomes for this poorly served subgroup of patients.

SECTION 2 – INCIDENCE AND EPIDEMIOLOGY

The incidence of melanoma has been rising steadily over the last 40 years, with a trend towards stabilisation in mortality except in elderly males.[7] Large geographic variations exist across countries and world regions, with the highest incidence rates observed in Australia and New Zealand (42/100 000 person-years for males and 31/100 000 person-years for females), followed by Western Europe (19/100 000 person-years for males and females), North America (18/100 000 person-years for males and 14/100 000 person-years for females) and Northern Europe (17/100 000 person-years for males and 18/100 000 person-years for females).[8]

Melanoma is rare in most African and Asian countries, with incidence rates commonly <1/100 000 person-years. Mortality rates peak at 5/100 000 person-years in New Zealand, although geographic variations in mortality rates are less pronounced than for incidence rates. The burden from melanoma is estimated to increase to 510 000 new cases (a roughly 50% increase) and to 96 000 deaths (a 68% increase) by 2040.[8]

UV irradiation has been identified as a major carcinogen involved in melanomagenesis.[9,10] UV irradiation is associated with a distinct DNA damage signature and a high rate of mutations per megabase.[11] Indeed, in The Cancer Genome Atlas (TCGA) melanoma programme, a UV signature (i.e. high fraction of C>T transitions at dyprimidines) was identified in >75% of melanoma samples profiled,[12] and among all cancer types profiled by the TCGA, melanoma was associated with the highest tumour (somatic) mutation burden overall.[13] In a randomised trial, prevention of UV exposure, including the regular use of sunscreen, has been shown to diminish the incidence of primary cutaneous melanomas in an Australian population.[14]

SECTION 3 – DIAGNOSIS AND PATHOLOGY/MOLECULAR BIOLOGY

Diagnosis

Suspicious lesions are usually characterised using the 'ABCD' rule:[15] **A**symmetry, **B**order irregularities, **C**olour heterogeneity, **D**ynamics (dynamics or evolution in colours, elevation or size). Nodular melanoma (NM) may lack the aforementioned characteristics. Here, the elevated, firm and growing (EFG) rule should be used, prompting excision.[16]

The 'ugly duckling' concept helps to identify melanomas, because naevi in the same individual tend to resemble one another and melanomas often do not fit the individual's naevus pattern.[17]

Dermoscopy by an experienced physician enhances the diagnostic accuracy.[18] An automated videodermoscopy system can provide improved diagnostic accuracy for patients with multiple atypical naevi during follow-up. Full body imaging with high resolution pictures has also been shown to improve early detection.[19]

Machine-learning algorithms trained on either standard or dermoscopic images have been shown to correctly diagnose pigmented skin lesions with a success rate comparable to that of a panel of 21 board-certified dermatologists; however, their use in clinical practice has not been evaluated.[20] Currently, there is insufficient data on the use of computer-assisted diagnostic tools for the detection of keratinocyte cancers and, as such, these cannot be widely recommended.[21]

Diagnosis should be based on a full-thickness complete excisional biopsy with a minimal margin (1-2 mm) of clinically uninvolved skin. Processing of the primary tumour according to international guidelines and by an experienced pathology institute is mandatory.

The histology report should follow the eighth edition of the American Joint Committee on Cancer (AJCC8) TNM (tumour–node–metastases) melanoma staging system,[22,23] as shown in **Supplementary Table S2**, and include:[22-25]

i) anatomical site;
ii) degree of sun damage of surrounding skin;
iii) cell of derivation;

iv) the diagnosis and clinico-pathological subtype [superficial spreading melanoma, NM, lentigo maligna melanoma (LMM), acral lentiginous and desmoplastic melanoma]; if the malignant nature of the lesion is uncertain, this should be stated in the report;

v) immunohistochemistry in cases where histological diagnosis is unclear [S-100 protein, Melan-A, human melanoma black (HMB)45, SRY-related HMG-box (SOX)10, MIB-1];

vi) the maximum thickness in millimetres (Breslow) reported to the nearest 0.1 mm (rounding up starting at 0.05);

vii) presence or absence of ulceration;

viii) presence and extent or absence of regression;

ix) presence or absence of microsatellites;

x) lymphovascular invasion;

xi) growth phase (horizontal or vertical);

xii) neurotropism/perineural invasion;

xiii) TILs;

xiv) presence of lymphatic emboli;

xv) mitotic rate. Although removed as a staging criterion for T1 tumours in AJCC8, it remains an overall important prognostic factor that should be reported for all patients with T1-T4 primary cutaneous melanoma;

xvi) presence or absence of tumour at the deep and peripheral edges of the biopsy.

Information on anatomical site (including extra-cutaneous sites such as mucosa and conjunctiva) and degree of sun damage of the surrounding skin is necessary. In rare situations, melanomas may derive from dermal melanocytes (melanoma arising from giant congenital naevus, malignant blue naevus and spitzoid), which should be reported as well.[26] Atypical spitzoid tumours should be distinguished from spitzoid melanoma as the former do not have metastatic potential. In these melanomas, the prognostic relevance of tumour thickness and sentinel lymph node (SLN) involvement is questionable.[27]

Mandatory and recommended information to include in a melanoma histopathology report is provided in **Supplementary Table S3**.

SECTION 4 – STAGING AND RISK ASSESSMENT

Staging and risk assessment procedures are determined by disease presentation at diagnosis.

Physical examination with special attention to any suspicious pigmented lesions, tumour satellites, in-transit metastases (ITMs) and regional lymph nodes (LNs) is mandatory.

In low-risk primary cutaneous melanomas (pT1a), no additional investigations are necessary. In higher tumour stages, ultrasound (US) for the detection of possible locoregional LN metastasis and/or computed tomography (CT) or positron emission tomography (PET) scans represent options to ensure proper tumour extension assessment before surgical treatment and staging with sentinel lymph node biopsy (SLNB). For patients with disease stage IIB or higher, brain magnetic resonance imaging (MRI) should be carried out.

SLNB is not routinely recommended for pT1a melanomas but can be discussed for special cases [e.g. 3 mitoses/mm^2, a positive deep margin or when Breslow thickness cannot be reliably determined (pTx)].[22] SLNB should be discussed with patients with a melanoma of AJCC8 stage pT1b (i.e. with a tumour thickness >0.8-1.0 mm or with a tumour thickness of <0.8 mm with ulceration). SLNB has no therapeutic impact but is recommended for all patients with clinically node negative stage T2a or higher melanoma (according to AJCC8), i.e. >1.0 mm Breslow thickness.

Carrying out an SLNB procedure should also take into consideration the risk–benefit for the individual patient, which includes assessment of patient age, comorbidities, performance status (PS), contraindications for systemic therapy and compliance, patient preference and potential morbidity.[28] This is particularly important for patients with *BRAF*-mutated, thick primary stage IIB or IIC melanoma since, for these patients, in case of a positive SLNB, the patient would be classed as having stage III melanoma and could be offered targeted therapy. For patients with T3b, T4a or T4b melanoma who qualify for adjuvant therapy, omitting SLNB can be discussed with the patient, but the potential benefits of SLNB in terms of staging and locoregional control should also be discussed.

SLNB should only be carried out in experienced centres. Quality control criteria for these centres include the following:[29]

- Histological review of the primary tumour should include immunohistochemical assessment.
- Marking of the primary tumour excision scar should be carried out during the consultation, preferably with photo documentation.
- SLNB and wide local excision (WLE) should be carried out by an experienced surgical team.
- SLNB procedures should be carried out simultaneously with the WLE to avoid lymph drainage modifications.
- Lymphoscintigraphy, including single-photon emission computed tomography (SPECT) imaging, should be carried out prior to WLE and SLNB to increase the accuracy of SLNB.
- SPECT imaging technique is particularly recommended for melanomas of the head and neck.
- Histological assessment of the of the SLNB should be according to cell morphology and should include step sectioning and immunohistochemical analysis. Comparison with the primary tumour histology is recommended in difficult cases.

The AJCC8 TNM melanoma staging system, which includes SLN staging information (which was required for inclusion in T2 or greater melanomas and optional for T1 melanomas to be included in analyses that informed revisions to the staging system) is the recommended classification system (see **Supplementary Table S2**, available online).[22,23,30]

In the setting of an isolated ITM or local recurrence of a primary melanoma without clinically or radiographically evident regional nodal or distant metastases, SLNB can be discussed if not carried out previously.

In the Multicentre Selective Lymphadenectomy Trial I (MSLT-I), there was no significant treatment-related difference between the WLE/SLNB versus WLE/nodal observation arms in the 10-year follow-up melanoma-specific survival rate in patients with intermediate-thickness melanomas (1.2-3.5 mm) and thick primary melanomas (>3.5 mm).[31] A criticised subgroup analysis appeared to show a significant benefit in favour of patients with node-positive disease in the SLNB arm compared with those in the observation arm. However, any patients with false-positive results (i.e. node-negative disease) or false-positive SLNB results were not considered. A further statistical method to prove this was developed using the interim data but it was not externally validated and therefore should not be used.[32,33]

In summary, findings from MSLT-I validated the staging potential of SLNB but did not show any unequivocal survival benefit and SLNB should therefore not be considered as a therapeutic procedure.

SLN tumour burden has been assessed in different ways, but all of them show that it adds to the prognostic accuracy.[34] The most commonly used and reproducible method between pathologists is the maximum diameter of the largest lesion (MDLL) according to the Rotterdam Criteria, which the European Organisation for Research and Treatment of Cancer (EORTC) has validated and adopted.[35] An MDLL cut-off of 1 mm has been used for adjuvant therapy trials. Therefore, use of the EORTC/Rotterdam Criteria for reporting SLN tumour burden is recommended. The AJCC, College of American Pathologists (CAP) and the International Collaboration on Cancer Reporting (ICCR) recommend measurement and recording of the largest maximum dimension (measured in millimeters to the nearest 0.1 mm using an ocular micrometer) of the largest discrete metastatic melanoma deposit in sentinel nodes.[22,23,36]

Complete lymph node dissection (CLND) for patients with SLN-positive disease is no longer the standard of care (SoC). After MSLT-I, two further trials [MSLT-II and the German Dermatologic Cooperative Oncology Group-selective lymphadenectomy Trial (DeCOG-SLT)] evaluated the benefit of carrying out routine CLND for SLN-positive disease. Both trials reported no impact on survival for early CLND compared with nodal observation with periodic US of the SLN-positive basin.[37,38] CLND provides additional staging information as approximately 20% of patients with SLN-positive disease have additional non-SLN involvement. However, upstaging occurs even less frequently

(approximately 6% of cases). Thus, considering the morbidity of routine CLND, this practice can no longer be recommended.[39-41]

SECTION 5 – MANAGEMENT OF LOCAL/LOCOREGIONAL DISEASE

Adjuvant radiotherapy

Findings from a prospective randomised trial demonstrated that adjuvant irradiation after LN dissection reduces the risk of relapse in the irradiation field by ~50% but has no impact on recurrence-free survival (RFS) and overall survival (OS).[42] Since local control is rarely the therapeutic objective in melanoma, adjuvant radiotherapy (RT) can no longer be routinely recommended. However, RT for local tumour control can be considered in cases of inadequate resection margins of lentigo maligna. RT can also be discussed for patients who have had a resection of melanoma metastases with microscopic tumour at the margin (R1) (only when additional surgery is not adequate) or after resection of bulky disease.[43]

SECTION 6 – MANAGEMENT OF ADVANCED/METASTATIC DISEASE

Melanoma brain metastases

Over 20% of real-world patients have evidence of central nervous system (CNS) metastases at diagnosis of unresectable disease.[44] Management of melanoma brain metastases (MBMs) is particularly challenging as brain involvement usually dictates a negative outcome for patients with melanoma, with symptomatic brain metastases (BMs) also associated with potentially serious effects on patient quality of life. Therefore, these patients need interdisciplinary care in specialised referral centres. Multidisciplinary decision-making should be based on the number of BMs, location, size and presence of oedema, along with presence and extension of extracranial disease.[45]

A proposed algorithm for the management of patients with MBMs is provided in **Figure 5**.

Clinical trials have confirmed that the approved systemic therapies for advanced melanoma can be safely and efficiently applied in patients with MBMs. Thus, four modalities and their combinations should be considered and applied depending on the individuals' needs: neurosurgery, stereotactic radiosurgery (SRS), targeted therapy with a BRAF inhibitor (BRAFi)–MEK inhibitor (MEKi) combination and immunotherapies. Whole

brain radiotherapy (WBRT) should be avoided whenever possible due to its lack of evidence for efficacy. The optimal sequence or combination of local and systemic therapies has not been determined, but recent results can help with decision-making until ongoing clinical trials bring more definitive answers.

Dabrafenib–trametinib was investigated in a prospective, multicentre, multicohort, open-label, phase II clinical trial (COMBI-MB) of patients with an Eastern Cooperative Oncology Group (ECOG) PS of 0 or 1.[46] In this trial, the response rate (RR) of 58% seen in asymptomatic patients with previously untreated MBMs was similar to the RRs reported in other organ sites. However, the median intracranial PFS was only 5.6 months.

Immunotherapy with anti-PD-1 monotherapy or ipilimumab–nivolumab has been evaluated in patients with MBMs. In the randomised, phase II ABC trial, outcomes were in favour of the combination.[47] The 5-year OS rates for therapy-naïve patients with asymptomatic MBMs were of 51% in the ipilimumab–nivolumab group versus 34% in the nivolumab group, with an even greater difference observed among patients who had not received prior treatment with BRAF–MEK inhibitors. Corresponding intracranial RRs were 51% and 20%, respectively. For previously-treated patients or those with symptomatic MBMs or leptomeningeal disease (LMD), the 5-year OS rate was 13% and the intracranial RR was 6%.[48]

In CheckMate 204, 165 screened patients were enrolled and treated with upfront ipilimumab–nivolumab: 101 patients were asymptomatic (cohort A; median follow-up 34.3 months) and 18 were symptomatic and could have received up to 4 mg dexamethasone or equivalent (cohort B; median follow-up 7.5 months). In cohort A, the 3-year intracranial PFS rate was 54.1% and the OS rate was 71.9%. In cohort B, the 3-year intracranial PFS rate was 18.9% and the OS rate was 36.6%. The durable 3-year response, OS and PFS rates reported in this trial support the first-line use of nivolumab–ipilimumab in patients with asymptomatic MBMs.[49] Patients with symptomatic MBMs have a poorer outcome, but some still could derive benefit from this therapy; however, the majority (61%) of patients with symptomatic MBMs developed immediate intracranial disease progression, indicating the need for upfront local treatment for these patients.

In the phase III NIBIT-M2 trial, 76 patients with active, untreated and asymptomatic MBMs were randomised to receive either fotemustine (Arm A), ipilimumab–fotemustine (Arm B) or ipilimumab–nivolumab (Arm C). At a median follow-up of 67 months, median OS was 8.5, 8.2 and 29.2 months for Arms A, B and C, respectively. The 7-year OS rate was 10.9%, 10.3% and 42.8%, respectively.[50]

It is worth noting that the strict entry criteria for these trials resulted in the inclusion of patients with a low CNS tumour burden. Nevertheless, based on these data, ipilimumab–nivolumab should be considered as the preferred first-line systemic treatment approach for patients with asymptomatic MBMs regardless of *BRAF* mutation status.

In the multicentre, open-label, single-arm, phase II TRICOTEL trial, patients with previously untreated metastatic melanoma (previous stereotactic RT was allowed), CNS metastases of ≥5 mm and an ECOG PS of ≤2 were treated with immunotherapy in combination with targeted therapy in two cohorts according to tumour *BRAF* mutation status. Patients with *BRAF* V600-wild type (WT) melanoma received i.v. atezolizumab–cobimetinib and those with *BRAF* V600-mutated melanoma received i.v. atezolizumab–vemurafenib–cobimetinib; continuation of systemic therapy was allowed in patients developing oligoprogressive MBMs treated with stereotactic RT. The *BRAF* V600-WT cohort was closed early after enrolment of 15 patients, with intracranial ORR determined by investigator assessment only in this cohort. Intracranial ORR was 42% (95% CI 29% to 54%) in the *BRAF* V600-mutated cohort (independent assessment) and 27% (95% CI 8% to 55%) in the *BRAF* V600-WT cohort (investigator assessment). These data therefore suggest that the combination of atezolizumab–vemurafenib–cobimetinib provide intracranial activity in patients with *BRAF* V600-mutated melanoma and CNS metastases.[51]

The optimal timing and sequencing of immune checkpoint inhibitor (ICI) and stereotactic RT or SRS remain unknown, although there are retrospective data suggesting that early SRS with concurrent ICI may be associated with improved efficacy[52] without an increased risk of radionecrosis.[53] Upfront SRS should be considered in particular for patients with symptomatic MBMs, those requiring treatment with steroids who are deemed eligible for local treatment and patients with high-risk MBMs, defined by their size, location and presence of peritumoral oedema. Since multiple sessions of SRS can be carried out at

different time points of the disease course, close monitoring using MRI is recommended so that SRS can be added when indicated.

Patients with MBMs in whom local therapy is not an option, those who have neurological symptoms requiring steroids (>10 mg prednisolone/day or equivalent) or those with LMD have a poor prognosis. Patients with *BRAF*-mutated melanoma can receive BRAFi–MEKi and, in the absence of further evidence, patients with *BRAF*-wild-type melanoma can be treated with ipilimumab–nivolumab (based on findings from CheckMate 204[49]) or chemotherapy (temozolomide, fotemustine, other). Nevertheless, given the poor prognosis of this patient population, the risk–benefit ratio of systemic anticancer therapy should be discussed carefully. In terms of RT, palliation with WBRT can be discussed for these patients, although data suggest that efficacy is limited and its use is discouraged in frail patients. For patients with LMD, findings from a recent phase II trial suggest that treatment with proton craniospinal irradiation (pCSI) may also be in option. In this trial, 63 patients with LMD from solid tumours (non-small cell lung and breast cancer) were randomised to receive either pCSI or photon involved-field RT (IFRT). The trial also included an exploratory cohort of patients with LMD from other solid tumours (n = 35, six of whom had melanoma) treated with pCSI. In the main analysis, compared with IFRT, pCSI was associated with improved median CNS PFS (7.5 versus 2.3 months, $P < 0.001$) and OS (9.9 versus 6.0 months, $P = 0.029$). The median CNS PFS and OS in the pCSI-treated exploratory cohort was 5.8 and 6.6 months, respectively.[54] Additional options for patients with LMD include intrathecal nivolumab and RT. Evidence supporting the use of intrathecal nivolumab are derived from a phase I trial which showed that one cycle of intrathecal nivolumab 50 mg followed by intravenous nivolumab 240 mg for subsequent cycles was associated with a median OS of 41 weeks and a 1-year OS rate of 43%.[55,56] Further guidance on the use of RT for MBMs can be found in the American Society for Radiation Oncology Clinical Practice Guideline.[57] Nevertheless, for this group of patients, palliative care must be discussed and activated.

Predictive and prognostic biomarkers

Simple clinical and laboratory parameters, including ECOG PS, lactate dehydrogenase (LDH) levels and the number of metastatic sites, offer predictive and prognostic value in patients undergoing targeted therapy. A pooled analysis of clinical trial data in the

advanced disease setting showed that patients with elevated LDH levels, more than three metastatic sites, and an ECOG PS of ≥1 had unfavourable outcomes when treated with BRAF–MEK inhibitors.[58]

As for PD-1 based immunotherapy, lower LDH levels and the absence of metastases beyond soft tissue and lung were identified as independent baseline characteristics associated with a favourable OS.[59] Predictive markers of efficacy for ICIs have been studied, from intracellular interactions in the tumour microenvironment (TME) and systemic markers, including: i) markers associated with tumour cells [e.g. programmed death-ligand 1 (PD-L1) expression, tumour mutational burden, specific mutated gene pathways], ii) TME (e.g. PD-L1 expression, tumour infiltrating immune cells), iii) circulating factors (e.g. peripheral blood cells, circulating tumour DNA, cytokines, inflammatory factors) and iv) host characteristics and markers (e.g. intestinal microbiome, human leukocyte antigen subtype, other specific mutations).

Some clinical parameters could also provide interesting biomarkers to select patients with the greatest benefit from ICI-based combination therapy. For example, in treatment-naive patients with asymptomatic MBMs, nivolumab–ipilimumab demonstrated a 59% intracranial RR compared with 21% for nivolumab. The 5-year OS rate also favoured the combination (55% versus 40%).[47,48] Elevated LDH is also associated with better outcomes with nivolumab–ipilimumab over single-agent nivolumab; PFS and OS HRs were 0.69 and 0.73, respectively, favouring nivolumab–ipilimumab in patients with LDH >2x the upper limit of normal.[60] PD-L1 expression is a potential biomarker and the 1% cut-off could provide a basis for discussing therapy with anti-PD-1 therapy alone or in combination with other ICIs. However, receiver operating characteristic curve analyses show that PD-L1 only marginally enriches the prediction compared with random assignment, thereby arguing against its value.[47,60,61]

Currently, no potential predictive biomarkers of efficacy for ICIs have been validated for clinical use.

SECTION 7 – SELF-EXAMINATION, RISK ASSESSMENT AND FOLLOW-UP

Self-examination and risk assessment

Patients with melanoma should be provided with instructions regarding the avoidance of sunburn or extended unprotected solar and/or artificial UV exposure, and lifelong regular self-examinations of the skin and peripheral LNs. Patients must be made aware that family members have an increased risk of developing melanoma. There is no recommendation for genetic testing. However, patients may be considered for genetic counselling and possibly genetic testing for melanoma if they have one or more of the following:[62]

- Three or more invasive melanomas, especially if one melanoma was diagnosed before 45 years of age.
- Three or more blood relatives on one side of the family who have had melanoma or cancer of the pancreas.
- Two or more Spitz nevi.
- One or more Spitz nevi and a close blood relative that has (or had) mesothelioma, meningioma or melanoma of the eye.

Follow-up

Proposals regarding follow-up are intended to improve the early detection of new melanomas but do not reduce the risk of occurrence of additional melanomas or improve the prognosis of patients with an already-detected melanoma.

There is currently no consensus regarding the frequency of follow-up examinations or the use of imaging techniques and blood tests for patients with resected melanoma. Recommendations vary from follow-up visits every 3 months during the first 3 years and every 6-12 months thereafter, to no organised follow-up at all. The authors encourage consultation of the respective national guidelines and adjustment, as required, considering available resources. Intervals between follow-up visits may be tailored according to individual risk and personal needs of the patient.[63]

During melanoma follow-up, patients should be clinically monitored in order to detect a relapse and to recognise additional skin tumours, especially secondary melanomas, as

early as possible.[17] However, it is not known whether this strategy leads to improved survival rates, especially in this new era of systemic therapies for stages II, III and IV disease. Eight percent of all patients with melanoma develop a secondary melanoma within 2 years of their initial diagnosis.[64] They also have an increased risk for other skin tumours. In patients with LMM, 35% of patients develop another cutaneous malignancy within five years.[43]

In the MSLT-II trial, patients who were assigned to the observation group were monitored by clinical examination every 4 months during the first 2 years, every 6 months during years 3-5 and then annually. Nodal ultrasonographic assessment of the sentinel node basin occurred at each visit for the first 5 years.

As patients with a thin primary melanoma (stage IA) have only a small risk of relapse, routine imaging techniques are not recommended for this patient population. In patients with stage IB or higher melanoma, or following treatment of metastases, US of LNs, CT or whole-body PET/PET-CT and brain MRI scans may lead to an earlier diagnosis of regional or systemic relapses.[65] The impact of radiological assessments on survival has not currently been demonstrated.[66] However, targeted therapy and immunotherapy demonstrate favourable effects in patients with a low tumour burden, which can be identified by high-resolution imaging during follow-up. LDH levels may be used for follow-up, although blood tests are not generally recommended.

A suggested follow-up schedule according to disease stage is proposed in **Figure 6**.

TABLES

Supplementary Table S1. Key clinical trials in melanoma that have included patients with MM

Trial	Phase	Stage and treatment setting	Therapy	Total number of patients (number of patients with MM)	Overall outcome [primary end point(s)]	Outcome in MM
SWOG S1801[67]	II	Resectable stage IIIB-IVC Neoadjuvant	Neoadjuvant pembrolizumab 200 mg Q3W 3 doses, surgery, adjuvant pembrolizumab 200 mg Q3W 15 doses versus surgery followed by adjuvant pembrolizumab 200 mg Q3W 18 doses	313 (4)	2-yr EFS 72% versus 49%	NA

CheckMate 238[68,69]	III	Resectable stage IIIB, IIIC or IV Adjuvant	Nivolumab 3 mg/kg Q2W for 1 yr versus ipilimumab 10 mg/kg Q3W 4 doses then Q12W up to 1 yr	906 (29)	4-yr RFS 51.7% versus 41.2% (HR 0.71, 95% CI 0.60-0.86)	4-yr RFS HR 1.71 (95% CI 0.68-4.29)
RELATIVITY-047[3,70]	II/III	Unresectable stage III or IV First line	Nivolumab 480mg and relatlimab 160 mg Q4W versus nivolumab 480mg Q4W	714 (51)	Median PFS 10.2 versus 4.6 mo (HR 0.78; 95% CI 0.64-0.94)	Median PFS HR 0.64 (95% CI 0.32-1.25)
Post hoc analysis[71]	KEYNOTE-001: Ib KEYNOTE-002: II KEYNOTE-006: III	Unresectable stage III or IV KEYNOTE-001: ipilimumab–[PD-(L)1 inhibitor]-naive or treated	Pembrolizumab 2 mg/kg Q3W	1567 (84)	Non-MM: Median PFS 4.2 mo Median OS 23.5 mo ORR 33%	Median PFS: 2.8 mo Median OS 11.3 mo ORR 19%

		KEYNOTE-002: ipilimumab-refractory<br>KEYNOTE-006: ipilimumab-naive				
Pooled analysis[6]	CA209-003: I<br>CA209-038: I<br>CheckMate 066: III<br>CheckMate 037: III<br>CheckMate 067: III	Unresectable stage III/IV	Nivolumab monotherapy<br><br>Nivolumab–ipilimumab<br><br>Ipilimumab monotherapy	889 (86)<br><br>407 (35)<br><br>357 (36)	Cutaneous: ORR 40.9% Median PFS 6.2 mo<br><br>Cutaneous: ORR 60.4% Median PFS 11.7 mo<br><br>Cutaneous: ORR 21.2% Median PFS 3.9 mo	ORR 23.3% Median PFS 3.0 mo<br><br>ORR 37.1% Median PFS 5.9 mo<br><br>ORR 8.3% Median PFS 2.7 mo

KEYNOTE-041[72]	Ib	Advanced or metastatic 0-2 prior lines of therapy for advanced disease	Pembrolizumab 2 mg/kg Q3W	42 (8)	ORR 24.3%	ORR 25.0%
POLARIS-01[73]	II	Locally advanced or metastatic After failure of ≥1 prior systemic therapy	Toripalimab 3 mg/kg Q2W	128 (22)	ORR 17.3% per RECIST v1.1	ORR 0% per RECIST v1.1
KEYNOTE-151[74]	Ib	Locally advanced or metastatic After failure of first-line systemic therapy	Pembrolizumab 2 mg/kg Q3W	103 (15)	ORR 16.7%	ORR 13.3%

CheckMate 172[75]	II	Advanced Patients progressing on or after ipilimumab	Nivolumab 3 mg/kg Q2W	1008 (63)	Median OS (non-acral cutaneous melanoma) 25.3 mo	Median OS 11.5 mo
C-144-01[4,5]	II	Advanced After progression on anti-PD-1-based therapy	Lifileucel	153 (15)	ORR 31.4%	ORR 50%

CI, confidence interval; EFS, event-free survival; HR, hazard ratio; MM, mucosal melanoma; mo, months; NA, not available; ORR, objective response rate; OS, overall survival; PD-1, programmed cell death protein 1; PD-L1, programmed death-ligand 1; PFS, progression-free survival; Q12W, every 12 weeks; Q2W, every 2 weeks; Q3W, every 3 weeks; Q4W, every 4 weeks; RECIST, Response Evaluation Criteria in Solid Tumours; RFS, recurrence-free survival; yr, year.

Supplementary Table S2. AJCC staging system for melanoma (eighth edition)[22,23]

**T – primary tumour**		
**T category**	**Thickness (mm)**	**Ulceration**
TX primary tumour thickness cannot be assessed (e.g. diagnosis by courettage)	NA	NA
T0 no evidence of primary tumour (e.g. unknown primary or completely regressed melanoma)	NA	NA
Tis (melanoma *in situ*)	NA	NA
T1	≤1.0	Unknown or unspecified
T1a	<0.8	Without ulceration
T1b	<0.8<br>0.8-1.0	With ulceration<br>With or without ulceration
T2	>1.0-2.0	Unknown or unspecified
T2a	>1.0-2.0	Without ulceration
T2b	>1.0-2.0	With ulceration
T3	>2.0-4.0	Unknown or unspecified
T3a	>2.0-4.0	Without ulceration

T3b	>2.0-4.0	With ulceration
T4	>4.0	Unknown or unspecified
T4a	>4.0	Without ulceration
T4b	>4.0	With ulceration
**N – lymph nodes**		
**N category**	**Number of tumour-involved regional LNs**	**Presence of in-transit, satellite and/or microsatellite metastases**
NX	Regional nodes not assessed (e.g. SLNB not carried out, regional nodes previously removed for another reason)	No
N0	No regional metastases detected	No
N1	One tumour-involved node or in-transit, satellite and/or microsatellite metastases with no tumour-involved nodes	
N1a	One clinically occult (i.e. detected by SLNB)	No
N1b	One clinically detected	No
N1c	No regional lymph node disease	Yes

N2	Two or three tumour-involved nodes or in-transit, satellite and/or microsatellite metastases with one tumour-involved node	
N2a	Two or three clinically occult (i.e. detected by SLNB)	No
N2b	Two or three, at least one of which was clinically detected	No
N2c	One clinically occult or clinically detected	Yes
N3	Four or more tumour-involved nodes or in-transit, satellite and/or microsatellite metastases with two or more tumour-involved nodes, or any number of matted nodes without or with in-transit, satellite and/or microsatellite metastases	
N3a	Four or more clinically occult (i.e. detected by SLNB)	No
N3b	Four or more, at least one of which was clinically	No

	detected, or presence of any number of matted nodes	
N3c	Two or more clinically occult or clinically detected and/or presence of any number of matted nodes	Yes
**M – metastasis**		
**M category**	**Anatomic site**	**LDH level**
M0	No evidence of distant metastasis	NA
M1	Evidence of distant metastasis	See below
M1a	Distant metastasis to skin, soft tissue including muscle and/or non-regional LN	Not recorded or unspecified
M1a(0)		Not elevated
M1a(1)		Elevated
M1b	Distant metastasis to lung with or without M1a sites of disease	Not recorded or unspecified
M1b(0)		Not elevated
M1b(1)		Elevated
M1c	Distant metastasis to non-CNS visceral sites with or without M1a or M1b sites of disease	Not recorded or unspecified
M1c(0)		Not elevated
M1c(1)		Elevated
M1d		Not recorded or unspecified

M1d(0)	Distant metastasis to CNS with or without M1a, M1b or M1c sites of disease	Normal
M1d(1)		Elevated

**pTNM**			
**Pathological stage group**	**T**	**N**	**M**
0	Tis	N0	M0
IA	T1a	N0	M0
IA	T1b	N0	M0
IB	T2a	N0	M0
IIA	T2b	N0	M0
IIA	T3a	N0	M0
IIB	T3b	N0	M0
IIB	T4a	N0	M0
IIC	T4b	N0	M0
IIIB	T0	N1b, N1c	M0
IIIC	T0	N2b/c, N3b/c	M0
IIIA	T1a/b, T2a	N1a, N2a	M0
IIIB	T1a/b, T2a	N1b/c, N2b	M0
IIIB	T2b, T3a	N1a/b/c, N2a/b	M0
IIIC	T1a/b, T2a/b, T3a	N2c, N3a/b/c	M0

IIIC	T3b, T4a	Any N ≥N1	M0
IIIC	T4b	N1a/b/c, N2a/b/c	M0
IIID	T4b	N3a/b/c	M0
IV	Any T, Tis	Any N	M1

Suffixes for M category: (0) LDH no elevated; (1) LDH elevated; no suffix is used if LDH is not recorded or is unspecified.

Pathological stage 0 and pathological stage T1 without clinically detected regional or distant metastases (pTis/pT1 cN0 cM0) do not require pathological evaluation of LNs to complete pathological staging; use cN0 to assign pathological stage.

AJCC, American Joint Committee on Cancer; CNS, central nervous system; LDH, lactate dehydrogenase; LN, lymph node; M, metastasis; N, node; NA, not applicable; p, pathological; SLNB, sentinel lymph node biopsy; T, tumour.

Reprinted from Gershenwald et al.[22] with permission by Springer International Publishing.

Supplementary Table S3. Mandatory and recommended information to include in a melanoma histopathology report[25]

Information to be included	Optional	Recommended	Mandatory
Anatomical site	X		
Degree of sun damage of surrounding skin	X		
Cell of derivation	X		
Diagnosis and clinico-pathological subtype (SSM, NM, LMM, ALM and DM)<br><br>If the malignant nature of the lesion is uncertain, this should be stated in the report			X
Immunohistochemistry in cases where histological diagnosis is unclear (S-100 protein, Melan-A, HMB45 and SOX10 for the confirmation of the melanocytic nature of the tumour; HMB45 as an additional feature of malignancy when there is an inverted positive gradient; MIB-1 as a proliferation marker)		X	
The maximum tumour thickness in millimetres (Breslow) reported to the nearest 0.1 mm (rounding up starting at 0.05)			X
Presence or absence of ulceration			X

Mitotic rate (although removed as a staging criterion for T1 tumours in AJCC8, it remains an overall important prognostic factor that should be reported for all patients with T1-T4 primary cutaneous melanoma)		X	
Presence or absence of microsatellites, defined as any discontinuous nest of intra-lymphatic metastatic cells of >0.05 mm in diameter clearly separated by normal dermis or subcutaneous fat from the invasive component of the tumor by a distance of at least 0.3 mm			X
Presence or absence of tumour at the deep and peripheral edges of the biopsy			X
Growth phase (horizontal or vertical)		X	
Presence and extent or absence of regression			X
TILs		X	
Presence of lymphatic emboli		X	
LVI		X	
Neurotropism/perineural invasion		X	

AJCC8, American Joint Committee on Cancer eighth edition; ALM, acral lentiginous melanoma; DM, desmoplastic melanoma; HMB, human melanoma black; LMM, lentigo maligna melanoma; LVI, lymphovascular invasion; NM, nodular melanoma; SOX, SRY-related HMG-box; SSM, superficial spreading melanoma; T, tumour; TIL, tumour-infiltrating lymphocyte.

Supplementary Table S4. *BRAF* mutations by class[76,77]

***BRAF* mutation class**	***BRAF* mutations**
Class I	*BRAF* V600E, *BRAF* V600K, *BRAF* V600D, *BRAF* V600M, *BRAF* V600R
Class II	*BRAF* L597Q/R/S/V, G464 V/E, G496A/V/R, K601E/N/T, P367 L/S
Class III	*BRAF* D594G, D594N, G466E, G466V

Supplementary Table S5. Biomarkers and molecular targets for precision medicines and corresponding ESCAT scores

Biomarker or genomic alteration	Method of detection	Drug match	ESCAT score[a,b]
BRAF	Sanger sequencing, pyrosequencing, PCR, NGS	BRAF inhibitors alone or in combination with MEK inhibitors	I-A[78-81]
NRAS	Sanger sequencing, pyrosequencing, PCR, NGS	MEK inihibitors	V[82]
c-KIT	Sanger sequencing, pyrosequencing, PCR, NGS	Kit inhibitors	V[83,84]
PD-L1 expression	IHC	Anti-LAG3 inhibitors	N/A

ESCAT, ESMO Scale for Clinical Actionability of molecular Targets; IHC, immunohistochemistry; MEK, mitogen-activated protein kinase kinase; N/A, not applicable; NGS, next-generation sequencing; PD-L1, programmed death-ligand 1.

[a]ESCAT scores apply to genomic alterations only. These scores have been defined by the guideline authors and assisted as needed by the ESMO Translational Research and Precision Medicine Working Group.[85]

[b]I-A, alteration–drug match is associated with improved outcome with evidence from randomised clinical trials showing the alteration–drug match in a specific tumour type results in a clinically meaningful improvement of a survival endpoint; V, alteration-drug match is associated with objective response, but without clinically meaningful benefit.[85]

Supplementary Table S6. Frequently mutated genes found in different melanoma subtypes[12,30]

**Melanoma subtype**	**Frequently mutated genes**
Cutaneous non-acral	*BRAF, CDKN2A, NRAS, TERT* promoter, *PTEN, TP53, NF1*
Acral	*BRAF, NRAS, NF1*
Mucosal	*NRAS*, *BRAF, NF1, KIT, SF3B1*

Supplementary Table S7. Local excision margins[86]

**Wide local excision margins according to AJCC8 melanoma staging system (pT1a-pT4b Nx M0)**	
**Tumour thickness (Breslow) in mm**	**Excision margin (cm)**
Melanoma *in situ* (pTis N0 M0)	0.5
<2 mm (pT1a-pT2 N0 M0)	1
>2 mm (pT3a-pT4b N0 M0)	2

AJCC8, American Joint Committee on Cancer eighth edition; M, metastasis; N, node; p, pathological; T, tumour; Tis, carcinoma *in situ*.

Reproduced from Thompson et al.[86] with permission.

Supplementary Table S8. Summary of stage subgroup eligibility criteria and RFS data for key adjuvant trials

		All patients had NED						
**Study**	**Drug regimen**	**IIB**	**IIC**	**IIIA**	**IIIB**	**IIIC**	**IIID**	**IV**
BRIM8[87] AJCC7	Vemurafenib versus placebo		HR NE	HR 0.52 (SLN >1 mm)	HR 0.63	HR 0.8		
COMBI-AD[88,89] AJCC7	Dabrafenib–trametinib versus placebo			HR 0.61 (SLN >1 mm)	HR 0.50	HR 0.48		
COMBI-AD[89] AJCC8 (*post hoc* analysis)	Dabrafenib–trametinib versus placebo			HR 0.83	HR 0.51	HR 0.50	HR 0.34	

EORTC 18071[90,91]<br><br>AJCC7	Ipilimumab 10 mg/kg versus placebo			HR 0.87 (SLN > 1 mm)	HR 0.81	HR 0.77 (1-3 LNs)<br><br>HR 0.64 (≥4 LNs)		
CheckMate 238[68,92,93]<br><br>AJCC7	Nivolumab versus ipilimumab 10 mg/kg				HR 0.72	HR 0.76		HR 0.64 (M1a,b)<br><br>HR 0.98 (M1c)
CheckMate 238[93]<br><br>AJCC8 (*post hoc* analysis)	Nivolumab versus ipilimumab 10 mg/kg				HR 0.58	HR 0.84	0.39	
KEYNOTE-054/EORTC 1325[94-96]	Pembrolizumab versus placebo			HR 0.66	HR 0.59	HR 0.61		

AJCC7								
KEYNOTE-054/EORTC 1325[96]<br><br>AJCC8 (*post hoc* analysis)	Pembrolizumab versus placebo			HR 0.72	HR 0.60	HR 0.53	HR 0.65	
KEYNOTE-716[97-99]<br><br>AJCC8	Pembrolizumab versus placebo	HR 0.60 (T3b)<br><br>HR 0.55 (T4a)	HR 0.65 (T4b)					
CheckMate 76K[100]<br><br>AJCC8	Nivolumab versus placebo	HR 0.34	HR 0.51					

All trials including patients with stage IIIA disease required a minimum SLN diameter of 1 mm. All patients with stage III disease included in these trials had undergone radical LN dissection.

AJCC7, American Joint Committee on Cancer seventh edition; AJCC8, American Joint Committee on Cancer eighth edition; HR, hazard ratio; LN, lymph node; M, metastasis; NE, not established; NED, no evidence of disease; RFS, recurrence-free survival; SLN, sentinel lymph node.

Supplementary Table S9. Definitions of best pathological response according to the INMC[101]

**Response**	**Definition**
pCR	Complete absence of viable tumour in the treated tumour bed
MPR or near pCR	>0%-≤10% of viable tumour in the treated tumour bed<br>Note: this may represent a meaningful end point in the context of neoadjuvant ICI therapy
pPR	>10%-≤50% of the treated tumour bed is occupied by viable tumour cells<br>Note: percent tumour regression associated with improved patient outcomes for both targeted therapy and immunotherapy is an area of active investigation
pNR	>50% or more of the treated tumour bed is occupied by viable tumour cells

ICI, immune checkpoint inhibitor; INMC, International Neoadjuvant Melanoma Consortium; MPR, major pathological response; pCR, pathological complete response; pNR, pathological non-response; pPR, pathological partial response.

Supplementary Table S10. ESMO-MCBS table for therapies/indications in cutaneous melanoma

Therapy	Disease setting	Trial	Control	Absolute survival gain	HR (95% CI)	QoL/toxicity	ESMO-MCBS v1.1 score[a]
**Neoadjuvant therapy**							
Neoadjuvant nivolumab–ipilimumab[b] (adjuvant therapy given to patients with a non-MPR after surgery)	Adult patients with resectable stage III melanoma	NADINA[102]<br>Phase III<br>NCT04949113	No neoadjuvant therapy (surgery followed by adjuvant nivolumab)<br>12-month EFS: 57.2%	EFS gain: 26.5%	EFS: 0.32 (0.15-0.66)[c]	QoL end point data not yet reported	A (Form 1)
**Adjuvant therapy**							
Dabrafenib–trametinib	Adult patients with Stage III melanoma with a *BRAF V600* mutation, following complete resection	COMBI-AD[88,89,103-105]<br>Phase III<br>NCT01682083	Placebo<br>Median RFS: 16.6 months<br>8-year OS: 65%	RFS gain: 76.5 months<br>8-year RFS gain: 6%	RFS: 0.52 (0.43-0.63)<br>OS: 0.80 (0.62-1.01), P = 0.06	QoL was an exploratory endpoint<br>≥25% discontinuation due to AEs	NEB (Form 1)[d]

Ipilimumab[e]	Patients with cutaneous melanoma with pathologic involvement of regional lymph nodes of >1 mm who have undergone complete resection, including total lymphadenectomy	EORTC 18071[106-108]<br><br>Phase III<br><br>NCT00636168	Placebo<br><br>Median RFS: 17.1 months<br><br>5-year OS: 54.4%	RFS gain: 10.5 months<br><br>5-year OS gain: 11.0%	RFS: 0.76 (0.64-0.89)<br><br>OS: 0.72 (0.58-0.88)[f]	No QoL benefit<br><br>>30% Grade 3-4 immune-related AEs<br><br>>10% premature discontinuation due to AEs (53% discontinued due to AEs in the ipilimumab arm, including 38.6% who discontinued within 12 weeks after randomisation)	A (Form 1)
Nivolumab<br><br>Stage III-IV disease	Adults and adolescents 12 years of age and older with Stage IIB or IIC melanoma, or melanoma with involvement of lymph nodes or metastatic disease who have undergone	CheckMate 238[69,93,109]<br><br>Phase III<br><br>NCT02388906	Ipilimumab<br><br>Median RFS: 24.1 months<br><br>5-year RFS: 39%	RFS gain: 36.9 months<br><br>5-year RFS gain: 11%	RFS: 0.72 (0.60-0.86)	Fewer treatment- related grade 3 or 4 AEs: 14.4% versus 45.9%<br><br>Fewer treatment discontinuation due to treatment-related AEs: 7.7% versus 41.7%<br><br>QoL data pending	NEB (Form 1)

	complete resection		5-year OS: 72%	5-year OS gain: 4%	OS: 0.86 (0.66-1.12)		
Nivolumab<br>Stage IIB-IIC disease	Adults and adolescents ≥12 years of age with Stage IIB or IIC melanoma, melanoma with involvement of lymph nodes or metastatic disease who have undergone complete resection	CheckMate 76K[100]<br>Phase III<br>NCT04099251	Placebo<br>Median RFS: NR<br>1-year RFS: 79.4%	RFS gain: NA<br>1-year RFS gain: 9.6%	RFS: 0.42 (0.30–0.59), *P* < 0.0001	QoL was not a study endpoint<br>Treatment-related grade 3 or 4 AEs: 10.3% versus 2.3%<br>Treatment discontinuation due to treatment-related AEs: 17.9% versus 2.7%	A (Form 1)
Pembrolizumab<br>Stage IIB/IIC disease	Adults and adolescents aged 12 years and older with Stage IIB, IIC or III melanoma and who have undergone	KEYNOTE-716[97,98]<br>Phase III<br>NCT03553836	Placebo<br>36-month RFS: 63.4%	36-month RFS gain: 12.8%	RFS: 0.62 (0.49-0.79)	QoL was an exploratory endpoint<br>Treatment discontinuation due to treatment-related AEs: 15.9% versus 2.5%	A (Form 1)

	complete resection						
Pembrolizumab<br>Stage III disease	Adults and adolescents aged 12 years and older with Stage IIB, IIC or III melanoma and who have undergone complete resection	KEYNOTE-054[95,96,110,111]<br>Phase III<br>NCT02362594	Placebo<br>5-year RFS: 38.3%	5-year RFS gain: 17.1%	RFS: 0.61 (0.51-0.72)	Treatment discontinuation due to treatment-related AEs: 13.8% versus 2.2%	A (Form 1)
**Metastatic**							
**First-line targeted therapy (*BRAF* V600-mutated)**							
Trametinib	Adult patients with unresectable or metastatic melanoma with a *BRAF V600* mutation	METRIC[112-114]<br>Phase III<br>NCT01245062	Dacarbazine or paclitaxel<br>Median PFS: 1.5 months	PFS gain: 3.4 months	PFS: 0.54 (0.41-0.73)	Improved QoL	4 (Form 2b)
Vemurafenib	Adult patients with *BRAF* V600E	BRIM-3[115,116]	Dacarbazine			QoL was not a prespecified endpoint	4 (Form 2a)

BRAF V600E mutation	mutation-positive unresectable or metastatic melanoma	Phase III<br><br>NCT01006980	Median PFS: 1.6 months<br><br>Median OS: 10.0 months	PFS gain: 5.3 months<br><br>OS gain: 3.3 months	PFS: 0.39 (0.33-0.47)<br><br>OS: 0.75 (0.60-0.93)		
Vemurafenib[g]<br><br>*BRAF* V600 *mutation*	Adult patients with *BRAF* V600 (E or K) mutation-positive unresectable or metastatic melanoma	BRIM-3[115-117]<br><br>Phase III<br><br>NCT01006980	Dacarbazine<br><br>Median PFS: 1.6 months<br><br>Median OS: 9.7 months	 <br>PFS gain: 5.3 months<br><br>OS gain: 3.9 months	 <br>PFS: 0.38 (0.32-0.46)<br><br>OS: 0.81 (0.67-0.98)	QoL was not a prespecified endpoint	4 (Form 2a)
Atezolizumab–cobimetinib–vemurafenib[e]	Patients with *BRAF* V600 mutation-positive unresectable or metastatic melanoma	IMspire150[118,119]<br><br>Phase III<br><br>NCT02908672	Placebo–cobimetinib–vemurafenib<br><br>Median PFS: 10.6 months<br><br>Median OS: 25.8 months	 <br>PFS gain: 4.5 months<br><br>OS gain: 13.2 months	 <br>PFS: 0.78 (0.63-0.97)	QoL data pending	3 (Form 2b)

					OS: 0.84 (0.66-1.06), *P* = 0.14		
Binimetinib–encorafenib	Adult patients with unresectable or metastatic melanoma with a *BRAF V600* mutation	COLUMBUS[81, 120-123]<br><br>Phase III<br><br>NCT01909453	Vemurafenib<br><br>Median PFS: 7.3 months<br><br>Median OS: 16.9 months<br><br>7-year OS: 18.2%	PFS gain: 7.6 months<br><br>OS gain: 16.7 months<br><br>7-year OS gain: 9.2%	PFS: 0.51 (0.39-0.66)<br><br>OS: 0.67 (0.53-0.84)	Improved QoL	A/5 (Form 1 & 2a)
Cobimetinib–vemurafenib	Adult patients with unresectable or metastatic melanoma with a *BRAF V600* mutation	coBRIM[78,124-127]<br><br>Phase III<br><br>NCT01689519	Placebo–vemurafenib<br><br>Median PFS: 7.2 months<br><br>Median OS: 17.4 months	PFS gain: 5.1 months<br><br>OS gain: 5.1 months	PFS: 0.58 (0.46-0.72)<br><br>OS: 0.80 (0.64-0.99)	9% reduction in skin cancer<br><br>2.8% increase in grade 3+ retinopathy<br><br>QoL was not a prespecified endpoint	A/5 (Form 1 & 2a)

			5-year OS: 26%	5-year OS gain: 5%			
Dabrafenib[h]	Adult patients with unresectable or metastatic melanoma with a *BRAF V600* mutation	BREAK-3[128-130]<br><br>Phase III<br><br>NCT01227889	Dacarbazine<br><br>Median PFS: 2.7 months	PFS gain: 2.4 months	PFS: 0.30 (0.18-0.51)	Improved QoL	4 (Form 2b)
Dabrafenib–trametinib	Adult patients with unresectable or metastatic melanoma with a *BRAF V600* mutation	COMBI-d[131-135]<br><br>Phase III<br><br>NCT01584648	Dabrafenib–placebo<br><br>Median PFS: 8.8 months<br><br>Median OS: 18.7 months<br><br>3-year OS: 32%	PFS gain: 2.2 months<br><br>OS gain: 6.4 months<br><br>3-year OS gain: 12%	PFS: 0.67 (0.53-0.84)<br><br>OS: 0.71 (0.55-0.92)	6% reduction in skin cancer<br><br>QoL was an exploratory endpoint	5 (Form 2a)

Dabrafenib–trametinib	Adult patients with unresectable or metastatic melanoma with a *BRAF V600* mutation	COMBI-v[135-138]<br><br>Phase III<br><br>NCT01597908	Vemurafenib<br><br>Median OS: 17.2 months<br><br>3-year OS: 31.0%	OS gain: 7.7 months[i]<br><br>3-year OS gain: 13.0%	Interim OS: 0.69 (0.53-0.89)	16% reduction in skin cancer<br>3% increase in grade ≥3 pyrexia<br>QoL was an exploratory endpoint	5 (Form 2a)
**First-line immunotherapy**							
Ipilimumab–dacarbazine	Advanced (unresectable or metastatic) melanoma in adults and adolescents 12 years of age and older	CA184-024[139,140]<br><br>Phase III<br><br>NCT00324155	Placebo–dacarbazine<br><br>Median OS: 9.1 months<br><br>2-year OS: 17.8%<br><br>5-year OS: 8.8%	OS gain: 2.1 months<br><br>2-year OS gain: 11.1%<br><br>5-year OS gain: 9.4%	OS: 0.69 (0.57-0.84)	QoL was not a prespecified endpoint	A/4 (Form 1 & 2a)

Nivolumab	Advanced (unresectable or metastatic) melanoma in adults	CheckMate 066[141-144]<br><br>Phase III<br><br>NCT01721772	Dacarbazine<br><br>Median OS: 11.2 months<br><br>5-year OS: 17%	OS gain: 26.1 months<br><br>5-year OS gain: 22%	OS: 0.50 (0.40-0.63)	QoL data not eligible for evaluation due to low completion rates	A/4 (Form 1 & 2a)
Nivolumab	Advanced (unresectable or metastatic) melanoma in adults	CheckMate 067[145-151]<br><br>Phase III<br><br>NCT01844505	Ipilimumab<br><br>Median OS: 19.9 months<br><br>10-year OS: 19%	OS gain: 17.0 months<br><br>10-year OS gain: 18% (with plateau)	OS: 0.63 (0.52-0.76)	No QoL benefit	A/4 (Form 1 & 2a)
Nivolumab–ipilimumab	Advanced (unresectable or metastatic) melanoma in adults	CheckMate 067[145-151]<br><br>Phase III	Ipilimumab<br><br>Median OS: 19.9 months	OS gain: 52.0 months	OS: 0.53 (0.44-0.65)	No QoL benefit	A/4 (Form 1 & 2a)

		NCT01844505	10-year OS: 19%	10-year OS gain: 24% (with plateau)			
Pembrolizumab	Adults and adolescents aged 12 years and older with advanced (unresectable or metastatic) melanoma	KEYNOTE-006[152-155]<br><br>Phase III<br><br>NCT01866319<br><br>KEYNOTE-587[156]<br><br>Phase III<br><br>NCT03486873	Ipilimumab<br><br>Median OS: 15.9 months<br><br>10-year OS: 23.6%	OS gain: 16.8 months[j]<br><br>10-year OS gain: 10.4%[j]	OS: 0.71 (0.60-0.85)	QoL was an exploratory endpoint	A/4 (Form 1 & 2a)
Relatlimab–nivolumab[k]	Adult and paediatric patients 12 years of age or older with	RELATIVITY-047[3,70,157]	Nivolumab			QoL was an exploratory endpoint	3 (Form 2b)

	unresectable or metastatic melanoma	Phase II/III<br><br>NCT03470922	Median PFS: 4.63 months	PFS gain: 5.59 months	PFS: 0.78 (0.64-0.94)		
**Intralesional therapy for locally advanced unresectable melanoma**							
T-VEC	Adults with unresectable melanoma that is regionally or distantly metastatic (stage IIIB, IIIC and IVM1a) with no bone, brain, lung or other visceral disease	OPTiM[158,159]<br><br>Phase III<br><br>NCT00769704	GM-CSF<br><br>Median OS: 18.9 months	 <br>OS gain: 4.4 months	 <br>OS: 0.79 (0.62-1.00), P = 0.0494	QoL was not a prespecified endpoint	3 (Form 2a)
**Second-line therapy**							
Ipilimumab[l]	Advanced (unresectable or metastatic) melanoma in adults and adolescents 12	MDX010-20[160,161]<br><br>Phase III	Glycoprotein 100<br><br>Median OS: 6.4 months	 <br>OS gain: 3.7 months	 <br>OS: 0.66 (0.51-0.87)	No QoL benefit	4 (Form 2a)

	years of age and older	NCT00094653					
Pembrolizumab[l]<br><br>2 mg/kg	Adults and adolescents aged 12 years and older with advanced (unresectable or metastatic) melanoma	KEYNOTE-002[162-164]<br><br>Phase II<br><br>NCT01704287	Investigator's choice of ChT<br><br>Median PFS: 2.7 months	PFS gain: 0.2 months<br><br>PFS gain >10% at 2 years with plateau	PFS: 0.57 (0.45-0.73)	QoL was an exploratory endpoint<br><br>Reduced grade 3-4 toxicity impacting on daily well-being (including fatigue, pruritus, nausea, decreased appetite, diarrhoea, vomiting, constipation and asthenia: 2.2% versus 11.7%)	3 (Form 2b)
Pembrolizumab[l]<br><br>10 mg/kg	Adults and adolescents aged 12 years and older with advanced (unresectable or metastatic) melanoma	KEYNOTE-002[162-164]<br><br>Phase II<br><br>NCT01704287	Investigator's choice of ChT<br><br>Median PFS: 2.7 months	PFS gain: 0.2 months<br><br>PFS gain >10% at 2 years with plateau	PFS: 0.50 (0.39-0.64) with plateau	QoL was an exploratory endpoint<br><br>Reduced grade 3-4 toxicity impacting on daily well-being (including fatigue, pruritus, nausea, decreased appetite, diarrhoea, vomiting, constipation and asthenia: 5.0% versus 11.7%)	3 (Form 2b)

AE, adverse event; CI, confidence interval; ChT, chemotherapy; EFS, event-free survival; EMA, European Medicines Agency; ESMO-MCBS, ESMO-Magnitude of Clinical Benefit Scale; EORTC, European Organisation for the Research and Treatment of Cancer; FDA, Food and Drug Administration; GM-CSF, granulocyte macrophage colony-stimulating factor; HR, hazard ratio; MPR, major pathological response; mut, mutation; NA, not available; NEB, no evaluable benefit; NR, not reached; OS, overall survival; PD-L1, programmed death-ligand 1; PFS, progression-free survival; QoL, quality of life; RFS, recurrence-free survival; T-VEC, talimogene laherparepvec; WG, working group.

[a]ESMO-MCBS v1.1.[165] was used to calculate scores for therapies/indications approved by the EMA or FDA. The scores have been calculated and verified by the ESMO-MCBS WG and reviewed by the authors (https://www.esmo.org/guidelines/esmo-mcbs/esmo-mcbs-evaluation-forms).

[b]Not EMA or FDA approved as neoadjuvant therapy.

[c]99.9% CI.

[d]In an unplanned, *post hoc* analysis, the OS advantage was restricted to patients with *BRAF* V600E-mutated tumours (HR 0.75, 95% CI 0.58-0.96), and among patients with *BRAF* V600K-mutated tumours, there was a deleterious effect on OS (HR 1.95, 95% CI 0.84-4.50).

[e]FDA approved, not EMA approved.

[f]95.1% CI.

[g]EMA approved, not FDA approved for V600Kmut.

[h]EMA approved, FDA approved for V600Emut only.

[i]Estimated from Kaplan-Meier plot.

[j]10-year follow-up data are based on the KEYNOTE-587 extension study, designed to collect long-term efficacy data. 211 (63%) of 333 KEYNOTE-006 patients which were on follow-up at the end of the study agreed to participate to the extension study.

[k]FDA approved; EMA approved for PD-L1 expression <1%. EMA approval for PD-L1 expression <1% was based on an exploratory analysis demonstrating a PFS gain of 3.5 months (HR 0.68, 95% CI 0.53-0.86). This exploratory analysis does not meet the ESMO-MCBS requirements for subgroups (i.e. many subgroups and without adjustment) and so cannot be scored.

[l]EMA and FDA approvals are for any line of treatment, but this study was conducted in previously-treated patients so is shown in the second-line treatment section of this table.

Supplementary Table S11. Levels of evidence and grades of recommendation (adapted from the Infectious Diseases Society of America-United States Public Health Service Grading System[a])

Levels of evidence

I	Evidence from at least one large randomised, controlled trial of good methodological quality (low potential for bias) or meta-analyses of well-conducted randomised trials without heterogeneity
II	Small randomised trials or large randomised trials with a suspicion of bias (lower methodological quality) or meta-analyses of such trials or of trials demonstrated heterogeneity
III	Prospective cohort studies
IV	Retrospective cohort studies or case-control studies
V	Studies without control group, case reports, expert opinions

Grades of recommendation

A	Strong evidence for efficacy with a substantial clinical benefit, strongly recommended
B	Strong or moderate evidence for efficacy but with a limited clinical benefit, generally recommended
C	Insufficient evidence for efficacy or benefit does not outweigh the risk or the disadvantages (adverse events, costs, etc.), optional
D	Moderate evidence against efficacy or for adverse outcome, generally not recommended
E	Strong evidence against efficacy or for adverse outcome, never recommended

[a]Reprinted by permission of Oxford University Press on behalf of the Infectious Diseases Society of America.[166]

FIGURES

Supplementary Figure S1. Diagnostic work-up of cutaneous melanoma

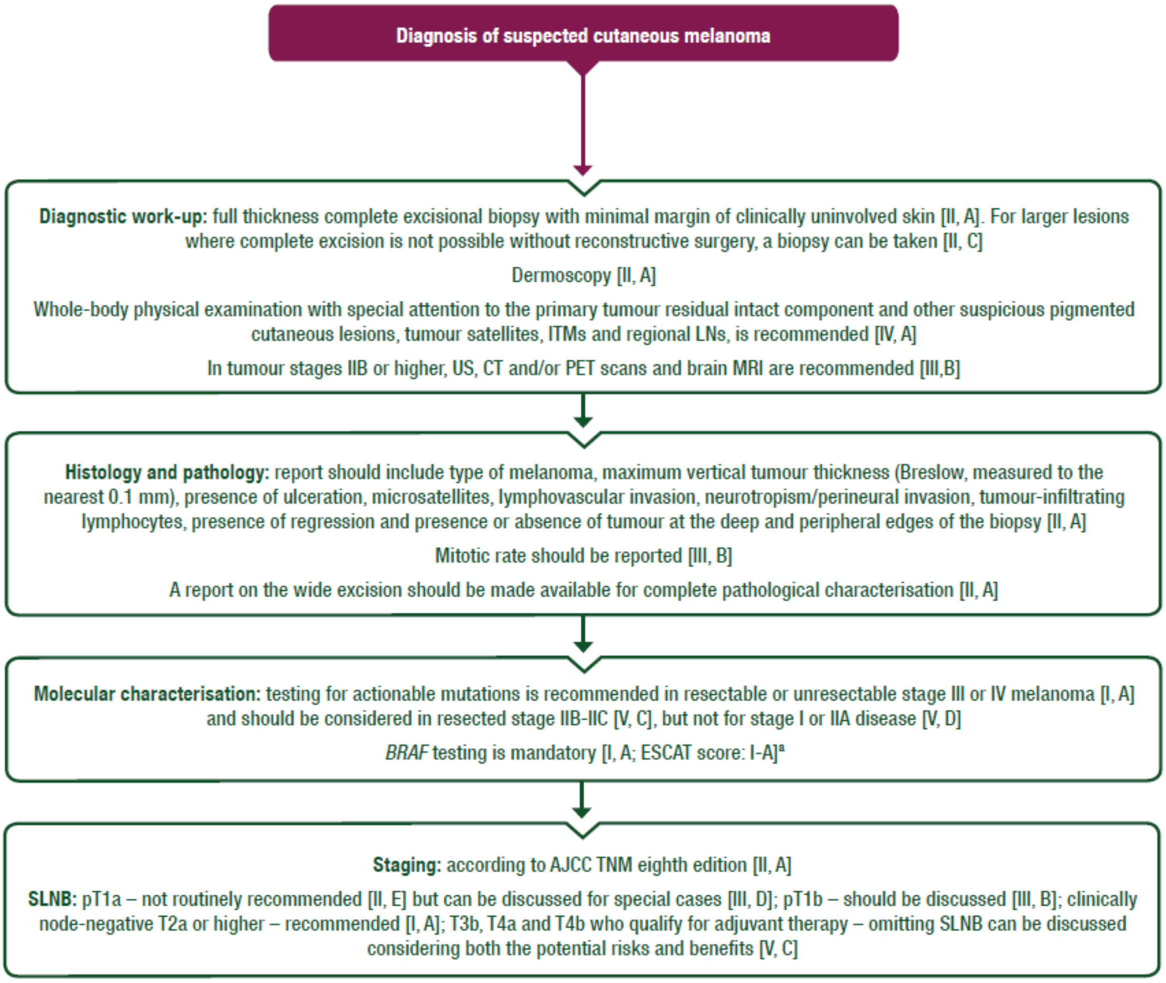

AJCC, American Joint Committee on Cancer; CT, computed tomography; ESCAT, ESMO Scale for Clinical Actionability of molecular Targets; ITM, in-transit metastasis; LN, lymph node; MRI, magnetic resonance imaging; PET, positron emission tomography; SLNB, sentinel lymph node biopsy; TNM, tumour–node–metastases; US, ultrasound.

[a]ESCAT scores apply to genomic alterations only. These scores have been defined by the guideline authors and assisted as needed by the ESMO Translational Research and Precision Medicine Working Group.[85]

REFERENCES

1. Tyrrell H, Payne M. Combatting mucosal melanoma: recent advances and future perspectives. *Melanoma Manag.* 2018;5(3):Mmt11.
2. Fortuna A, Amaral T. Multidisciplinary approach and treatment of acral and mucosal melanoma. *Front Oncol.* 2024;14:1340408.
3. Long GV, Hodi FS, Lipson EJ, et al. Overall Survival and Response with Nivolumab and Relatlimab in Advanced Melanoma. *NEJM Evidence.* 2023;2(4):EVIDoa2200239.
4. Grigoleit GU, Kluger H, Thomas S, et al. 1086MO Lifileucel tumor-infiltrating lymphocyte (TIL) cell therapy in patients (pts) with advanced mucosal melanoma after progression on immune checkpoint inhibitors (ICI): Results from the phase II C-144-01 study. *Annals of Oncology.* 2023;34:S654.
5. Chesney J, Lewis KD, Kluger H, et al. Efficacy and safety of lifileucel, a one-time autologous tumor-infiltrating lymphocyte (TIL) cell therapy, in patients with advanced melanoma after progression on immune checkpoint inhibitors and targeted therapies: pooled analysis of consecutive cohorts of the C-144-01 study. *J Immunother Cancer.* 2022;10(12):e005755.
6. D'Angelo SP, Larkin J, Sosman JA, et al. Efficacy and Safety of Nivolumab Alone or in Combination With Ipilimumab in Patients With Mucosal Melanoma: A Pooled Analysis. *J Clin Oncol.* 2017;35(2):226-235.
7. Garbe C, Keim U, Gandini S, et al. Epidemiology of cutaneous melanoma and keratinocyte cancer in white populations 1943-2036. *Eur J Cancer.* 2021;152:18-25.
8. Arnold M, Singh D, Laversanne M, et al. Global Burden of Cutaneous Melanoma in 2020 and Projections to 2040. *JAMA Dermatol.* 2022;158(5):495-503.
9. El Ghissassi F, Baan R, Straif K, et al. A review of human carcinogens--part D: radiation. *Lancet Oncol.* 2009;10(8):751-752.
10. World Health Organization. Artificial tanning devices: public health interventions to manage sunbeds. Available at https://www.who.int/publications/i/item/9789241512596. Published 2017. Accessed March 14, 2024.
11. Alexandrov LB, Nik-Zainal S, Wedge DC, et al. Signatures of mutational processes in human cancer. *Nature.* 2013;500:415.

12. Cancer Genome Atlas Network. Genomic Classification of Cutaneous Melanoma. *Cell.* 2015;161(7):1681-1696.
13. Lawrence MS, Stojanov P, Polak P, et al. Mutational heterogeneity in cancer and the search for new cancer-associated genes. *Nature.* 2013;499(7457):214-218.
14. Green AC, Williams GM, Logan V, et al. Reduced melanoma after regular sunscreen use: randomized trial follow-up. *J Clin Oncol.* 2011;29(3):257-263.
15. Friedman RJ, Rigel DS, Kopf AW. Early detection of malignant melanoma: the role of physician examination and self-examination of the skin. *CA Cancer J Clin.* 1985;35(3):130-151.
16. Chamberlain AJ, Fritschi L, Kelly JW. Nodular melanoma: patients' perceptions of presenting features and implications for earlier detection. *J Am Acad Dermatol.* 2003;48(5):694-701.
17. Grob JJ, Bonerandi JJ. The 'ugly duckling' sign: identification of the common characteristics of nevi in an individual as a basis for melanoma screening. *Arch Dermatol.* 1998;134(1):103-104.
18. Kittler H, Pehamberger H, Wolff K, et al. Diagnostic accuracy of dermoscopy. *Lancet Oncology.* 2002;3(3):159-165.
19. Salerni G, Carrera C, Lovatto L, et al. Benefits of total body photography and digital dermatoscopy ("two-step method of digital follow") in the early diagnosis of melanoma in patients at high risk for melanoma. *J Am Acad Dermatol.* 2012;67(1):e17-e27.
20. Esteva A, Kuprel B, Novoa RA, et al. Dermatologist-level classification of skin cancer with deep neural networks. *Nature.* 2017;542(7639):115-118.
21. Ferrante di Ruffano L, Takwoingi Y, Dinnes J, et al. Computer-assisted diagnosis techniques (dermoscopy and spectroscopy-based) for diagnosing skin cancer in adults. *Cochrane Database Syst Rev.* 2018;12(12):Cd013186.
22. Gershenwald JE, Scolyer RA, Hess KR, et al. Melanoma of the Skin In: Amin MB, Edge S, Greene F, et al., eds. *AJCC Cancer Staging Manual. 8th ed.* New York: Springer International Publishing; 2017:563-586.
23. Gershenwald JE, Scolyer RA, Hess KR, et al. Melanoma staging: Evidence-based changes in the American Joint Committee on Cancer eighth edition cancer staging manual. *CA Cancer J Clin.* 2017;67(6):472-492.

24. Long GV, Swetter SM, Menzies AM, et al. Cutaneous melanoma. *Lancet.* 2023;402(10400):485-502.
25. Garbe C, Amaral T, Peris K, et al. European consensus-based interdisciplinary guideline for melanoma. Part 1: Diagnostics: Update 2022. *Eur J Cancer.* 2022;170:236-255.
26. Whiteman DC, Pavan WJ, Bastian BC. The melanomas: a synthesis of epidemiological, clinical, histopathological, genetic, and biological aspects, supporting distinct subtypes, causal pathways, and cells of origin. *Pigment Cell Melanoma Res.* 2011;24(5):879-897.
27. Gassenmaier M, Soltanpour N, Held L, et al. Diagnostic and prognostic classification of atypical spitzoid tumours based on histology and genomic aberrations: A prospective cohort study with long-term follow-up. *Eur J Cancer.* 2022;163:200-210.
28. Han D, Zager JS, Shyr Y, et al. Clinicopathologic predictors of sentinel lymph node metastasis in thin melanoma. *J Clin Oncol.* 2013;31(35):4387-4393.
29. Dummer R, Ramelyte E, Levesque M, et al. Critical aspects to achieve a high-quality melanoma clinic. *Curr Opin Oncol.* 2017;29(2):145-150.
30. Hayward NK, Wilmott JS, Waddell N, et al. Whole-genome landscapes of major melanoma subtypes. *Nature.* 2017;545(7653):1-18.
31. Morton DL, Thompson JF, Cochran AJ, et al. Final trial report of sentinel-node biopsy versus nodal observation in melanoma. *N Engl J Med.* 2014;370(7):599-609.
32. Thomas JM. Prognostic false-positivity of the sentinel node in melanoma. *Nat Clin Pract Oncol.* 2008;5(1):18-23.
33. van Akkooi AC. Sentinel node followed by completion lymph node dissection versus nodal observation: staging or therapeutic? Controversy continues despite final results of MSLT-1. *Melanoma Res.* 2014;24(4):291-294.
34. van Akkooi ACJ, de Wilt JHW, Verhoef C, et al. Clinical relevance of melanoma micrometastases (<0.1 mm) in sentinel nodes: are these nodes to be considered negative? *Ann Oncol.* 2006;17(10):1578-1585.
35. van der Ploeg AP, van Akkooi AC, Haydu LE, et al. The prognostic significance of sentinel node tumour burden in melanoma patients: an international, multicenter study of 1539 sentinel node-positive melanoma patients. *Eur J Cancer.* 2014;50(1):111-120.

36. College of American Pathologists. Cancer Protocol Templates. Available at https://www.cap.org/protocols-and-guidelines/cancer-reporting-tools/cancer-protocol-templates. Accessed June 24, 2024.
37. Leiter U, Stadler R, Mauch C, et al. Complete lymph node dissection versus no dissection in patients with sentinel lymph node biopsy positive melanoma (DeCOG-SLT): a multicentre, randomised, phase 3 trial. *Lancet Oncol.* 2016;17(6):757-767.
38. Faries MB, Thompson JF, Cochran AJ, et al. Completion Dissection or Observation for Sentinel-Node Metastasis in Melanoma. *N Engl J Med.* 2017;376(23):2211-2222.
39. Madu MF, Franke V, Bruin MM, et al. Immediate completion lymph node dissection in stage IIIA melanoma does not provide significant additional staging information beyond EORTC SN tumour burden criteria. *Eur J Cancer.* 2017;87:212-215.
40. Verver D, van Klaveren D, van Akkooi ACJ, et al. Risk stratification of sentinel node-positive melanoma patients defines surgical management and adjuvant therapy treatment considerations. *Eur J Cancer.* 2018;96:25-33.
41. Coit D. The Enigma of Regional Lymph Nodes in Melanoma. *N Engl J Med.* 2017;376(23):2280-2281.
42. Burmeister BH, Henderson MA, Ainslie J, et al. Adjuvant radiotherapy versus observation alone for patients at risk of lymph-node field relapse after therapeutic lymphadenectomy for melanoma: a randomised trial. *Lancet Oncol.* 2012;13(6):589-597.
43. Farshad A, Burg G, Panizzon R, et al. A retrospective study of 150 patients with lentigo maligna and lentigo maligna melanoma and the efficacy of radiotherapy using Grenz or soft X-rays. *Br J Dermatol.* 2002;146(6):1042-1046.
44. Schina A, Pedersen S, Spenning AL, et al. Sustained improved survival of patients with metastatic melanoma after the introduction of anti-PD-1-based therapies. *Eur J Cancer.* 2023;195:113392.
45. Phillips WJ, Baghai T, Ong M, et al. A Contemporary Report of Clinical Outcomes in Patients with Melanoma Brain Metastases. *Curr Oncol.* 2021;28(1):428-439.
46. Davies MA, Saiag P, Robert C, et al. Dabrafenib plus trametinib in patients with BRAFV600-mutant melanoma brain metastases (COMBI-MB): a multicentre, multicohort, open-label, phase 2 trial. *Lancet Oncol.* 2017;18(7):863-873.

47. Long GV, Atkinson V, Lo S, et al. Combination nivolumab and ipilimumab or nivolumab alone in melanoma brain metastases: a multicentre randomised phase 2 study. *Lancet Oncol.* 2018;19(5):672-681.
48. Long GV, Atkinson V, Lo S, et al. Five-year overall survival from the anti-PD1 brain collaboration (ABC Study): Randomized phase 2 study of nivolumab (nivo) or nivo+ipilimumab (ipi) in patients (pts) with melanoma brain metastases (mets). *J Clin Oncol.* 2021;39(15_suppl):9508.
49. Tawbi HA, Forsyth PA, Hodi FS, et al. Long-term outcomes of patients with active melanoma brain metastases treated with combination nivolumab plus ipilimumab (CheckMate 204): final results of an open-label, multicentre, phase 2 study. *Lancet Oncol.* 2021;22(12):1692-1704.
50. Di Giacomo AM, Chiarion-Sileni V, Del Vecchio M, et al. Nivolumab plus ipilimumab in melanoma patients with asymptomatic brain metastases: 7-year outcomes and quality of life from the multicenter phase III NIBIT-M2 trial. *Eur J Cancer.* 2024;199:113531.
51. Dummer R, Queirolo P, Gerard Duhard P, et al. Atezolizumab, vemurafenib, and cobimetinib in patients with melanoma with CNS metastases (TRICOTEL): a multicentre, open-label, single-arm, phase 2 study. *Lancet Oncol.* 2023;24(12):e461-e471.
52. Lehrer EJ, Peterson J, Brown PD, et al. Treatment of brain metastases with stereotactic radiosurgery and immune checkpoint inhibitors: An international meta-analysis of individual patient data. *Radiother Oncol.* 2019;130:104-112.
53. Lehrer EJ, Kowalchuk RO, Gurewitz J, et al. Concurrent Administration of Immune Checkpoint Inhibitors and Stereotactic Radiosurgery is Not Associated with an Increased Risk of Radiation Necrosis: An International Multicenter Study of 657 Patients. *Int J Rad Oncol Biol Physics.* 2022;114(3; supplement):E58.
54. Yang JT, Wijetunga NA, Pentsova E, et al. Randomized Phase II Trial of Proton Craniospinal Irradiation Versus Photon Involved-Field Radiotherapy for Patients With Solid Tumor Leptomeningeal Metastasis. *J Clin Oncol.* 2022;40(33):3858-3867.
55. Glitza IC, Phillips S, John I, et al. 1082O Concurrent intrathecal (IT) and intravenous (IV) nivolumab (N) for melanoma (MM) patients (pts) with leptomeningeal disease (LMD). *Ann Oncol.* 2023;34:S652.

56. Glitza Oliva IC, Ferguson SD, Bassett R, Jr., et al. Concurrent intrathecal and intravenous nivolumab in leptomeningeal disease: phase 1 trial interim results. *Nat Med.* 2023;29(4):898-905.
57. Gondi V, Bauman G, Bradfield L, et al. Radiation Therapy for Brain Metastases: An ASTRO Clinical Practice Guideline. *Practical Radiation Oncology.* 2022;12(4):265-282.
58. Long GV, Grob JJ, Nathan P, et al. Factors predictive of response, disease progression, and overall survival after dabrafenib and trametinib combination treatment: a pooled analysis of individual patient data from randomised trials. *Lancet Oncol.* 2016;17(12):1743-1754.
59. Weide B, Martens A, Hassel JC, et al. Baseline Biomarkers for Outcome of Melanoma Patients Treated with Pembrolizumab. *Clin Cancer Res.* 2016;22(22):5487-5496.
60. Wolchok JD, Chiarion-Sileni V, Gonzalez R, et al. Overall Survival with Combined Nivolumab and Ipilimumab in Advanced Melanoma. *N Engl J Med.* 2017(377(14)):1345-1356.
61. Larkin J, Minor D, D'Angelo S, et al. Overall Survival in Patients With Advanced Melanoma Who Received Nivolumab Versus Investigator's Choice Chemotherapy in CheckMate 037: A Randomized, Controlled, Open-Label Phase III Trial. *J Clin Oncol.* 2018;36(4):383-390.
62. Leachman SA, Carucci J, Kohlmann W, et al. Selection criteria for genetic assessment of patients with familial melanoma. *J Am Acad Dermatol.* 2009;61(4):677.e671-614.
63. Turner RM, Bell KJL, Morton RL, et al. Optimizing the frequency of follow-up visits for patients treated for localized primary cutaneous melanoma. *J Clin Oncol.* 2011;29(35):4641-4646.
64. Titus-Ernstoff L, Perry AE, Spencer SK, et al. Multiple primary melanoma: two-year results from a population-based study. *Arch Dermatol.* 2006;142(4):433-438.
65. Bastiaannet E, Wobbes T, Hoekstra OS, et al. Prospective comparison of [18F]fluorodeoxyglucose positron emission tomography and computed tomography in patients with melanoma with palpable lymph node metastases: diagnostic accuracy and impact on treatment. *J Clin Oncol.* 2009;27(28):4774-4780.

66. Nieweg OE, Kroon BBR. The conundrum of follow-up: should it be abandoned? *Surg Oncol Clin N Am.* 2006;15(2):319-330.
67. Patel SP, Othus M, Chen Y, et al. Neoadjuvant-Adjuvant or Adjuvant-Only Pembrolizumab in Advanced Melanoma. *N Engl J Med.* 2023;388(9):813-823.
68. Weber J, Mandala M, Del Vecchio M, et al. Adjuvant Nivolumab versus Ipilimumab in Resected Stage III or IV Melanoma. *N Engl J Med.* 2017;377(19):1824-1835.
69. Ascierto PA, Del Vecchio M, Mandalá M, et al. Adjuvant nivolumab versus ipilimumab in resected stage IIIB-C and stage IV melanoma (CheckMate 238): 4-year results from a multicentre, double-blind, randomised, controlled, phase 3 trial. *Lancet Oncol.* 2020;21(11):1465-1477.
70. Tawbi HA, Schadendorf D, Lipson EJ, et al. Relatlimab and Nivolumab versus Nivolumab in Untreated Advanced Melanoma. *N Engl J Med.* 2022;386(1):24-34.
71. Hamid O, Robert C, Ribas A, et al. Antitumour activity of pembrolizumab in advanced mucosal melanoma: a post-hoc analysis of KEYNOTE-001, 002, 006. *British Journal of Cancer.* 2018;119(6):670-674.
72. Yamazaki N, Takenouchi T, Fujimoto M, et al. Phase 1b study of pembrolizumab (MK-3475; anti-PD-1 monoclonal antibody) in Japanese patients with advanced melanoma (KEYNOTE-041). *Cancer Chemother Pharmacol.* 2017;79(4):651-660.
73. Tang B, Chi Z, Chen Y, et al. Safety, Efficacy, and Biomarker Analysis of Toripalimab in Previously Treated Advanced Melanoma: Results of the POLARIS-01 Multicenter Phase II Trial. *Clinical Cancer Research.* 2020;26(16):4250-4259.
74. Si L, Zhang X, Shu Y, et al. A Phase Ib Study of Pembrolizumab as Second-Line Therapy for Chinese Patients With Advanced or Metastatic Melanoma (KEYNOTE-151). *Translational Oncology.* 2019;12(6):828-835.
75. Nathan P, Ascierto PA, Haanen J, et al. Safety and efficacy of nivolumab in patients with rare melanoma subtypes who progressed on or after ipilimumab treatment: a single-arm, open-label, phase II study (CheckMate 172). *European Journal of Cancer.* 2019;119:168-178.
76. Sahin IH, Klostergaard J. BRAF Mutations as Actionable Targets: A Paradigm Shift in the Management of Colorectal Cancer and Novel Avenues. *JCO Oncol Pract.* 2021;17(12):723-730.
77. Wan PTC, Garnett MJ, Roe SM, et al. Mechanism of Activation of the RAF-ERK Signaling Pathway by Oncogenic Mutations of B-RAF. *Cell.* 2004;116(6):855-867.

78. Larkin J, Ascierto PA, Dreno B, et al. Combined vemurafenib and cobimetinib in BRAF-mutated melanoma. *N Engl J Med.* 2014;371(20):1867-1876.
79. Robert C, Karaszewska B, Schachter J, et al. Improved overall survival in melanoma with combined dabrafenib and trametinib. *N Engl J Med.* 2015;372(1):30-39.
80. Johnson DB, Flaherty KT, Weber JS, et al. Combined BRAF (Dabrafenib) and MEK inhibition (Trametinib) in patients with BRAFV600-mutant melanoma experiencing progression with single-agent BRAF inhibitor. *J Clin Oncol.* 2014;32(33):3697-3704.
81. Dummer R, Ascierto PA, Gogas HJ, et al. Encorafenib plus binimetinib versus vemurafenib or encorafenib in patients with BRAF-mutant melanoma (COLUMBUS): a multicentre, open-label, randomised phase 3 trial. *Lancet Oncol.* 2018;19(5):603-615.
82. Dummer R, Schadendorf D, Ascierto PA, et al. Binimetinib versus dacarbazine in patients with advanced NRAS-mutant melanoma (NEMO): a multicentre, open-label, randomised, phase 3 trial. *Lancet Oncol.* 2017;18(4):435-445.
83. Hodi FS, Corless CL, Giobbie-Hurder A, et al. Imatinib for melanomas harboring mutationally activated or amplified KIT arising on mucosal, acral, and chronically sun-damaged skin. *J Clin Oncol.* 2013;31(26):3182-3190.
84. Janku F, Bauer S, Shoumariyeh K, et al. Efficacy and safety of ripretinib in patients with KIT-altered metastatic melanoma. *ESMO Open.* 2022;7(4):100520.
85. Mateo J, Chakravarty D, Dienstmann R, et al. A framework to rank genomic alterations as targets for cancer precision medicine: the ESMO Scale for Clinical Actionability of molecular Targets (ESCAT). *Ann Oncol.* 2018;29(9):1895-1902.
86. Thompson JF, Scolyer RA, Kefford RF. Cutaneous melanoma. *Lancet.* 2005;365(9460):687-701.
87. Maio M, Lewis K, Demidov L, et al. Adjuvant vemurafenib in resected, BRAFV600 mutation-positive melanoma (BRIM8): a randomised, double-blind, placebo-controlled, multicentre, phase 3 trial. *Lancet Oncol.* 2018;19(4):510-520.
88. Long GV, Hauschild A, Santinami M, et al. Adjuvant Dabrafenib plus Trametinib in Stage III BRAF-Mutated Melanoma. *N Engl J Med.* 2017;377(19):1813-1823.
89. Dummer R, Hauschild A, Santinami M, et al. Five-Year Analysis of Adjuvant Dabrafenib plus Trametinib in Stage III Melanoma. *N Engl J Med.* 2020;383(12):1139-1148.

90. Eggermont AMM, Chiarion-Sileni V, Grob J-J, et al. Prolonged Survival in Stage III Melanoma with Ipilimumab Adjuvant Therapy. *N Engl J Med.* 2016;375(19):1845-1855.
91. Eggermont AMM, Chiarion-Sileni V, Grob J-J, et al. Adjuvant ipilimumab versus placebo after complete resection of high-risk stage III melanoma (EORTC 18071): a randomised, double-blind, phase 3 trial. *Lancet Oncol.* 2015;16(5):522-530.
92. Weber JS, Mandalà M, Del Vecchio M, et al. Adjuvant therapy with nivolumab (NIVO) versus ipilimumab (IPI) after complete resection of stage III/IV melanoma: Updated results from a phase III trial (CheckMate 238). *J Clin Oncol.* 2018;36(15_suppl):9502.
93. Larkin J, Del Vecchio M, Mandalá M, et al. Adjuvant Nivolumab versus Ipilimumab in Resected Stage III/IV Melanoma: 5-Year Efficacy and Biomarker Results from CheckMate 238. *Clin Cancer Res.* 2023;29(17):3352-3361.
94. Eggermont AMM, Blank CU, Mandalà M, et al. Adjuvant Pembrolizumab versus Placebo in Resected Stage III Melanoma. *N Engl J Med.* 2018(378):1789-1801.
95. Eggermont AMM, Blank CU, Mandala M, et al. Longer Follow-Up Confirms Recurrence-Free Survival Benefit of Adjuvant Pembrolizumab in High-Risk Stage III Melanoma: Updated Results From the EORTC 1325-MG/KEYNOTE-054 Trial. *J Clin Oncol.* 2020;38(33):3925-3936.
96. Eggermont AMM, Kicinski M, Blank CU, et al. Five-Year Analysis of Adjuvant Pembrolizumab or Placebo in Stage III Melanoma. *NEJM Evidence.* 2022;1(11):EVIDoa2200214.
97. Luke JJ, Rutkowski P, Queirolo P, et al. Pembrolizumab versus placebo as adjuvant therapy in completely resected stage IIB or IIC melanoma (KEYNOTE-716): a randomised, double-blind, phase 3 trial. *Lancet.* 2022;399(10336):1718-1729.
98. Long GV, Luke JJ, Khattak MA, et al. Pembrolizumab versus placebo as adjuvant therapy in resected stage IIB or IIC melanoma (KEYNOTE-716): distant metastasis-free survival results of a multicentre, double-blind, randomised, phase 3 trial. *Lancet Oncol.* 2022;23(11):1378-1388.
99. Luke JJ, Ascierto PA, Khattak MA, et al. Pembrolizumab Versus Placebo as Adjuvant Therapy in Resected Stage IIB or IIC Melanoma: Final Analysis of Distant Metastasis-Free Survival in the Phase III KEYNOTE-716 Study. *J Clin Oncol.* 2024;42(14):1619-1624.

100. Kirkwood JM, Del Vecchio M, Weber J, et al. Adjuvant nivolumab in resected stage IIB/C melanoma: primary results from the randomized, phase 3 CheckMate 76K trial. *Nat Med.* 2023;29(11):2835-2843.
101. Tetzlaff MT, Messina JL, Stein JE, et al. Pathological assessment of resection specimens after neoadjuvant therapy for metastatic melanoma. *Ann Oncol.* 2018;29(8):1861-1868.
102. Blank CU, Lucas MW, Scolyer RA, et al. Neoadjuvant Nivolumab and Ipilimumab in Resectable Stage III Melanoma. *N Engl J Med.* 2024;391(18):1696-1708.
103. Hauschild A, Dummer R, Schadendorf D, et al. Longer Follow-Up Confirms Relapse-Free Survival Benefit With Adjuvant Dabrafenib Plus Trametinib in Patients With Resected BRAF V600-Mutant Stage III Melanoma. *J Clin Oncol.* 2018;36(35):3441-3449.
104. Schadendorf D, Hauschild A, Santinami M, et al. Patient-reported outcomes in patients with resected, high-risk melanoma with BRAF(V600E) or BRAF(V600K) mutations treated with adjuvant dabrafenib plus trametinib (COMBI-AD): a randomised, placebo-controlled, phase 3 trial. *Lancet Oncol.* 2019;20(5):701-710.
105. Long GV, Hauschild A, Santinami M, et al. Final Results for Adjuvant Dabrafenib plus Trametinib in Stage III Melanoma. *N Engl J Med.* 2024;391(18):1709-1720.
106. Eggermont AM, Chiarion-Sileni V, Grob JJ, et al. Prolonged Survival in Stage III Melanoma with Ipilimumab Adjuvant Therapy. *N Engl J Med.* 2016;375(19):1845-1855.
107. Coens C, Suciu S, Chiarion-Sileni V, et al. Health-related quality of life with adjuvant ipilimumab versus placebo after complete resection of high-risk stage III melanoma (EORTC 18071): secondary outcomes of a multinational, randomised, double-blind, phase 3 trial. *Lancet Oncol.* 2017;18(3):393-403.
108. Eggermont AM, Chiarion-Sileni V, Grob JJ, et al. Adjuvant ipilimumab versus placebo after complete resection of high-risk stage III melanoma (EORTC 18071): a randomised, double-blind, phase 3 trial. *Lancet Oncol.* 2015;16(5):522-530.
109. Weber J, Mandala M, Del Vecchio M, et al. Adjuvant Nivolumab versus Ipilimumab in Resected Stage III or IV Melanoma. *N Engl J Med.* 2017;377(19):1824-1835.
110. Eggermont AMM, Blank CU, Mandala M, et al. Adjuvant Pembrolizumab versus Placebo in Resected Stage III Melanoma. *N Engl J Med.* 2018;378(19):1789-1801.

111. Bottomley A, Coens C, Mierzynska J, et al. Adjuvant pembrolizumab versus placebo in resected stage III melanoma (EORTC 1325-MG/KEYNOTE-054): health-related quality-of-life results from a double-blind, randomised, controlled, phase 3 trial. *Lancet Oncol.* 2021;22(5):655-664.
112. Flaherty KT, Robert C, Hersey P, et al. Improved survival with MEK inhibition in BRAF-mutated melanoma. *N Engl J Med.* 2012;367(2):107-114.
113. Robert C, Flaherty K, Nathan P, et al. Five-year outcomes from a phase 3 METRIC study in patients with BRAF V600 E/K-mutant advanced or metastatic melanoma. *Eur J Cancer.* 2019;109:61-69.
114. Schadendorf D, Amonkar MM, Milhem M, et al. Functional and symptom impact of trametinib versus chemotherapy in BRAF V600E advanced or metastatic melanoma: quality-of-life analyses of the METRIC study. *Ann Oncol.* 2014;25(3):700-706.
115. Chapman PB, Hauschild A, Robert C, et al. Improved survival with vemurafenib in melanoma with BRAF V600E mutation. *N Engl J Med.* 2011;364(26):2507-2516.
116. McArthur GA, Chapman PB, Robert C, et al. Safety and efficacy of vemurafenib in BRAF(V600E) and BRAF(V600K) mutation-positive melanoma (BRIM-3): extended follow-up of a phase 3, randomised, open-label study. *Lancet Oncol.* 2014;15(3):323-332.
117. Chapman PB, Robert C, Larkin J, et al. Vemurafenib in patients with BRAFV600 mutation-positive metastatic melanoma: final overall survival results of the randomized BRIM-3 study. *Ann Oncol.* 2017;28(10):2581-2587.
118. Gutzmer R, Stroyakovskiy D, Gogas H, et al. Atezolizumab, vemurafenib, and cobimetinib as first-line treatment for unresectable advanced BRAF(V600) mutation-positive melanoma (IMspire150): primary analysis of the randomised, double-blind, placebo-controlled, phase 3 trial. *Lancet.* 2020;395(10240):1835-1844.
119. Ascierto PA, Stroyakovskiy D, Gogas H, et al. Overall survival with first-line atezolizumab in combination with vemurafenib and cobimetinib in BRAF(V600) mutation-positive advanced melanoma (IMspire150): second interim analysis of a multicentre, randomised, phase 3 study. *Lancet Oncol.* 2023;24(1):33-44.
120. Dummer R, Ascierto PA, Gogas HJ, et al. Overall survival in patients with BRAF-mutant melanoma receiving encorafenib plus binimetinib versus vemurafenib or

encorafenib (COLUMBUS): a multicentre, open-label, randomised, phase 3 trial. *Lancet Oncol.* 2018;19(10):1315-1327.

121. Dummer R, Flaherty KT, Robert C, et al. COLUMBUS 5-Year Update: A Randomized, Open-Label, Phase III Trial of Encorafenib Plus Binimetinib Versus Vemurafenib or Encorafenib in Patients With BRAF V600-Mutant Melanoma. *J Clin Oncol.* 2022:Jco2102659.
122. Gogas H, Dummer R, Ascierto PA, et al. Quality of life in patients with BRAF-mutant melanoma receiving the combination encorafenib plus binimetinib: Results from a multicentre, open-label, randomised, phase III study (COLUMBUS). *Eur J Cancer.* 2021;152:116-128.
123. Schadendorf D, Dummer R, Flaherty KT, et al. COLUMBUS 7-year update: A randomized, open-label, phase III trial of encorafenib plus binimetinib versus vemurafenib or encorafenib in patients with BRAF V600E/K-mutant melanoma. *Eur J Cancer.* 2024;204:114073.
124. Ascierto PA, McArthur GA, Dréno B, et al. Cobimetinib combined with vemurafenib in advanced BRAF(V600)-mutant melanoma (coBRIM): updated efficacy results from a randomised, double-blind, phase 3 trial. *Lancet Oncol.* 2016;17(9):1248-1260.
125. Dréno B, Ribas A, Larkin J, et al. Incidence, course, and management of toxicities associated with cobimetinib in combination with vemurafenib in the coBRIM study. *Ann Oncol.* 2017;28(5):1137-1144.
126. de la Cruz-Merino L, Di Guardo L, Grob JJ, et al. Clinical features of serous retinopathy observed with cobimetinib in patients with BRAF-mutated melanoma treated in the randomized coBRIM study. *J Transl Med.* 2017;15(1):146.
127. Ascierto PA, Dréno B, Larkin J, et al. 5-Year Outcomes with Cobimetinib plus Vemurafenib in BRAFV600 Mutation-Positive Advanced Melanoma: Extended Follow-up of the coBRIM Study. *Clin Cancer Res.* 2021;27(19):5225-5235.
128. Hauschild A, Grob JJ, Demidov LV, et al. Dabrafenib in BRAF-mutated metastatic melanoma: a multicentre, open-label, phase 3 randomised controlled trial. *Lancet.* 2012;380(9839):358-365.
129. Grob JJ, Amonkar MM, Martin-Algarra S, et al. Patient perception of the benefit of a BRAF inhibitor in metastatic melanoma: quality-of-life analyses of the BREAK-3 study comparing dabrafenib with dacarbazine. *Ann Oncol.* 2014;25(7):1428-1436.

130. Hauschild A, Ascierto PA, Schadendorf D, et al. Long-term outcomes in patients with BRAF V600-mutant metastatic melanoma receiving dabrafenib monotherapy: Analysis from phase 2 and 3 clinical trials. *Eur J Cancer.* 2020;125:114-120.
131. Long GV, Stroyakovskiy D, Gogas H, et al. Combined BRAF and MEK inhibition versus BRAF inhibition alone in melanoma. *N Engl J Med.* 2014;371(20):1877-1888.
132. Long GV, Stroyakovskiy D, Gogas H, et al. Dabrafenib and trametinib versus dabrafenib and placebo for Val600 BRAF-mutant melanoma: a multicentre, double-blind, phase 3 randomised controlled trial. *Lancet.* 2015;386(9992):444-451.
133. Long GV, Flaherty KT, Stroyakovskiy D, et al. Dabrafenib plus trametinib versus dabrafenib monotherapy in patients with metastatic BRAF V600E/K-mutant melanoma: long-term survival and safety analysis of a phase 3 study. *Ann Oncol.* 2017;28(7):1631-1639.
134. Schadendorf D, Amonkar MM, Stroyakovskiy D, et al. Health-related quality of life impact in a randomised phase III study of the combination of dabrafenib and trametinib versus dabrafenib monotherapy in patients with BRAF V600 metastatic melanoma. *Eur J Cancer.* 2015;51(7):833-840.
135. Schadendorf D, Long GV, Stroiakovski D, et al. Three-year pooled analysis of factors associated with clinical outcomes across dabrafenib and trametinib combination therapy phase 3 randomised trials. *Eur J Cancer.* 2017;82:45-55.
136. Robert C, Karaszewska B, Schachter J, et al. Improved overall survival in melanoma with combined dabrafenib and trametinib. *N Engl J Med.* 2015;372(1):30-39.
137. Robert C, Grob JJ, Stroyakovskiy D, et al. Five-Year Outcomes with Dabrafenib plus Trametinib in Metastatic Melanoma. *N Engl J Med.* 2019;381(7):626-636.
138. Grob JJ, Amonkar MM, Karaszewska B, et al. Comparison of dabrafenib and trametinib combination therapy with vemurafenib monotherapy on health-related quality of life in patients with unresectable or metastatic cutaneous BRAF Val600-mutation-positive melanoma (COMBI-v): results of a phase 3, open-label, randomised trial. *Lancet Oncol.* 2015;16(13):1389-1398.
139. Robert C, Thomas L, Bondarenko I, et al. Ipilimumab plus dacarbazine for previously untreated metastatic melanoma. *N Engl J Med.* 2011;364(26):2517-2526.

140. Maio M, Grob JJ, Aamdal S, et al. Five-year survival rates for treatment-naive patients with advanced melanoma who received ipilimumab plus dacarbazine in a phase III trial. *J Clin Oncol.* 2015;33(10):1191-1196.
141. Robert C, Long GV, Brady B, et al. Nivolumab in previously untreated melanoma without BRAF mutation. *N Engl J Med.* 2015;372(4):320-330.
142. Ascierto PA, Long GV, Robert C, et al. Survival Outcomes in Patients With Previously Untreated BRAF Wild-Type Advanced Melanoma Treated With Nivolumab Therapy: Three-Year Follow-up of a Randomized Phase 3 Trial. *JAMA Oncol.* 2019;5(2):187-194.
143. Robert C, Long GV, Brady B, et al. Five-Year Outcomes With Nivolumab in Patients With Wild-Type BRAF Advanced Melanoma. *J Clin Oncol.* 2020;38(33):3937-3946.
144. Long GV, Atkinson V, Ascierto PA, et al. Effect of nivolumab on health-related quality of life in patients with treatment-naïve advanced melanoma: results from the phase III CheckMate 066 study. *Ann Oncol.* 2016;27(10):1940-1946.
145. Larkin J, Chiarion-Sileni V, Gonzalez R, et al. Combined Nivolumab and Ipilimumab or Monotherapy in Untreated Melanoma. *N Engl J Med.* 2015;373(1):23-34.
146. Wolchok JD, Chiarion-Sileni V, Gonzalez R, et al. Overall Survival with Combined Nivolumab and Ipilimumab in Advanced Melanoma. *N Engl J Med.* 2017;377(14):1345-1356.
147. Hodi FS, Chiarion-Sileni V, Gonzalez R, et al. Nivolumab plus ipilimumab or nivolumab alone versus ipilimumab alone in advanced melanoma (CheckMate 067): 4-year outcomes of a multicentre, randomised, phase 3 trial. *Lancet Oncol.* 2018;19(11):1480-1492.
148. Larkin J, Chiarion-Sileni V, Gonzalez R, et al. Five-Year Survival with Combined Nivolumab and Ipilimumab in Advanced Melanoma. *N Engl J Med.* 2019;381(16):1535-1546.
149. Schadendorf D, Larkin J, Wolchok J, et al. Health-related quality of life results from the phase III CheckMate 067 study. *Eur J Cancer.* 2017;82:80-91.
150. Wolchok JD, Chiarion-Sileni V, Gonzalez R, et al. Long-Term Outcomes With Nivolumab Plus Ipilimumab or Nivolumab Alone Versus Ipilimumab in Patients With Advanced Melanoma. *J Clin Oncol.* 2022;40(2):127-137.

151. Wolchok JD, Chiarion-Sileni V, Rutkowski P, et al. Final, 10-Year Outcomes with Nivolumab plus Ipilimumab in Advanced Melanoma. *N Engl J Med.* 2024. https://doi.org/10.1056/NEJMoa2407417.
152. Robert C, Schachter J, Long GV, et al. Pembrolizumab versus Ipilimumab in Advanced Melanoma. *N Engl J Med.* 2015;372(26):2521-2532.
153. Schachter J, Ribas A, Long GV, et al. Pembrolizumab versus ipilimumab for advanced melanoma: final overall survival results of a multicentre, randomised, open-label phase 3 study (KEYNOTE-006). *Lancet.* 2017;390(10105):1853-1862.
154. Petrella TM, Robert C, Richtig E, et al. Patient-reported outcomes in KEYNOTE-006, a randomised study of pembrolizumab versus ipilimumab in patients with advanced melanoma. *Eur J Cancer.* 2017;86:115-124.
155. Robert C, Ribas A, Schachter J, et al. Pembrolizumab versus ipilimumab in advanced melanoma (KEYNOTE-006): post-hoc 5-year results from an open-label, multicentre, randomised, controlled, phase 3 study. *Lancet Oncol.* 2019;20(9):1239-1251.
156. Long GV, Carlino MS, McNeil C, et al. Pembrolizumab versus ipilimumab for advanced melanoma: 10-year follow-up of the phase III KEYNOTE-006 study. *Ann Oncol.* 2024. https://doi.org/10.1016/j.annonc.2024.08.2330.
157. Schadendorf D, Tawbi H, Lipson EJ, et al. Health-related quality of life with nivolumab plus relatlimab versus nivolumab monotherapy in patients with previously untreated unresectable or metastatic melanoma: RELATIVITY-047 trial. *Eur J Cancer.* 2023;187:164-173.
158. Andtbacka RH, Kaufman HL, Collichio F, et al. Talimogene Laherparepvec Improves Durable Response Rate in Patients With Advanced Melanoma. *J Clin Oncol.* 2015;33(25):2780-2788.
159. Andtbacka RHI, Collichio F, Harrington KJ, et al. Final analyses of OPTiM: a randomized phase III trial of talimogene laherparepvec versus granulocyte-macrophage colony-stimulating factor in unresectable stage III-IV melanoma. *J Immunother Cancer.* 2019;7(1):145.
160. Hodi FS, O'Day SJ, McDermott DF, et al. Improved survival with ipilimumab in patients with metastatic melanoma. *N Engl J Med.* 2010;363(8):711-723.

161. Revicki DA, van den Eertwegh AJ, Lorigan P, et al. Health related quality of life outcomes for unresectable stage III or IV melanoma patients receiving ipilimumab treatment. *Health Qual Life Outcomes.* 2012;10:66.
162. Ribas A, Puzanov I, Dummer R, et al. Pembrolizumab versus investigator-choice chemotherapy for ipilimumab-refractory melanoma (KEYNOTE-002): a randomised, controlled, phase 2 trial. *Lancet Oncol.* 2015;16(8):908-918.
163. Hamid O, Puzanov I, Dummer R, et al. Final analysis of a randomised trial comparing pembrolizumab versus investigator-choice chemotherapy for ipilimumab-refractory advanced melanoma. *Eur J Cancer.* 2017;86:37-45.
164. Schadendorf D, Dummer R, Hauschild A, et al. Health-related quality of life in the randomised KEYNOTE-002 study of pembrolizumab versus chemotherapy in patients with ipilimumab-refractory melanoma. *Eur J Cancer.* 2016;67:46-54.
165. Cherny NI, Dafni U, Bogaerts J, et al. ESMO-Magnitude of Clinical Benefit Scale version 1.1. *Ann Oncol.* 2017;28(10):2340-2366.
166. Dykewicz CA. Summary of the Guidelines for Preventing Opportunistic Infections among Hematopoietic Stem Cell Transplant Recipients. *Clinical Infectious Diseases.* 2001;33(2):139-144 (adapted from: Gross PA, Barrett TL, Dellinger EP, et al. Purpose of quality standards for infectious diseases. *Clin Infect Dis.*1994;18(3):421).
